# Evolution of IgE responses to multiple allergen components throughout childhood

**DOI:** 10.1016/j.jaci.2017.11.064

**Published:** 2018-10

**Authors:** Rebecca Howard, Danielle Belgrave, Panagiotis Papastamoulis, Angela Simpson, Magnus Rattray, Adnan Custovic

**Affiliations:** a Division of Informatics, Imaging and Data Sciences, Faculty of Biology, Medicine and Health, University of Manchester, Manchester, United Kingdom; b Section of Paediatrics, Department of Medicine, Imperial College London, London, United Kingdom; c Division of Infection, Immunity and Respiratory Medicine, University of Manchester and University Hospital of South Manchester, Manchester Academic Health Sciences Centre, Manchester, United Kingdom

**Keywords:** Allergens, asthma, childhood, component-resolved diagnostics, IgE, machine learning, rhinitis, CRD, Component-resolved diagnostics, HDM, House dust mite, ISAC, Immuno Solid-phase Allergen Chip, MCMC, Markov chain Monte Carlo algorithm, OR, Odds ratio, PR, Pathogenesis-related, SPT, Skin prick test

## Abstract

**Background:**

There is a paucity of information about longitudinal patterns of IgE responses to allergenic proteins (components) from multiple sources.

**Objectives:**

This study sought to investigate temporal patterns of component-specific IgE responses from infancy to adolescence, and their relationship with allergic diseases.

**Methods:**

In a population-based birth cohort, we measured IgE to 112 components at 6 follow-ups during childhood. We used a Bayesian method to discover cross-sectional sensitization patterns and their longitudinal trajectories, and we related these patterns to asthma and rhinitis in adolescence.

**Results:**

We identified 1 sensitization cluster at age 1, 3 at age 3, 4 at ages 5 and 8, 5 at age 11, and 6 at age 16 years. “Broad” cluster was the only cluster present at every follow-up, comprising components from multiple sources. “Dust mite” cluster formed at age 3 years and remained unchanged to adolescence. At age 3 years, a single-component “Grass” cluster emerged, which at age 5 years absorbed additional grass components and Fel d 1 to form the “Grass/cat” cluster. Two new clusters formed at age 11 years: “Cat” cluster and “PR-10/profilin” (which divided at age 16 years into “PR-10” and “Profilin”). The strongest contemporaneous associate of asthma at age 16 years was sensitization to dust mite cluster (odds ratio: 2.6; 95% CI: 1.2-6.1; *P* < .05), but the strongest early life predictor of subsequent asthma was sensitization to grass/cat cluster (odds ratio: 3.5; 95% CI: 1.6-7.4; *P* < .01).

**Conclusions:**

We describe the architecture of the evolution of IgE responses to multiple allergen components throughout childhood, which may facilitate development of better diagnostic and prognostic biomarkers for allergic diseases.

Allergic sensitization is a risk factor for asthma and rhinitis,^1^, ^2^, ^3^ but the strength of this association is inconsistent.^4^, ^5^ A patient is typically deemed to be sensitized based on a positive skin prick test (SPT) or a blood test measuring specific IgE to a range of common inhalant and food allergens.^6^, ^7^ However, both these tests can be positive without the patient having any symptoms,^8^ and neither positive SPT nor IgE can confirm the expression of symptoms on allergen exposure.^8^, ^9^ This is partly because the natural sources that are used to prepare the whole-allergen extracts for skin or blood testing contain multiple allergenic proteins (components), with each component potentially containing multiple epitopes for binding IgE.^10^ There is increasing evidence that sensitization to some, but not all of these proteins is important for the expression of allergic disease.^9^, ^11^, ^12^ Also, homologous proteins present in different allergen sources may be cross-reactive (eg, profilins and PR-10 proteins in various plants, or tropomyosin present in mites, insects, and crustaceans), and a positive SPT or IgE to whole allergen extract may reflect sensitization to a cross-reactive component.^12^, ^13^ Recent evidence suggests that assessing sensitization to allergen components (component-resolved diagnostics [CRD]) may be more informative than standard tests using whole allergen extracts.^14^

Current multiplex CRD platforms such as the Immuno Solid-phase Allergen Chip (ISAC) (ImmunoCAP ISAC; Thermo Fisher Scientific, Uppsala, Sweden) allow testing of small volumes of serum for component-specific IgE to more than 100 allergen components in a single assay,^13^, ^15^ with robust and reproducible results.^16^ We have previously shown that patterns of component-specific IgE responses in this multiplex chip-based assay have reasonable discrimination ability for asthma and rhinoconjuinctivitis.^17^ In a further study using latent variable modelling, we identified 3 cross-sectional clusters of IgE responses to different protein families at age 11 years, and each of these patterns was associated with different clinical symptoms.^18^ Our subsequent study has indicated that longitudinal trajectories of the cross-sectional sensitization patterns to a limited number of grass and house dust mite (HDM) allergens during childhood had different associations with clinical outcomes, suggesting that the time of onset of specific patterns of IgE response was critically important.^19^ Posa et al^20^ have recently shown that IgE polysensitization to several HDM molecules predicts current rhinitis and both current and future asthma.

Capturing the heterogeneity in longitudinal patterns of responses to multiple components from different sources is challenging, and the conventional analyses may overaggregate the underlying complexity.^21^ Cluster-based sensitization profiles may provide a methodological framework within which to address this issue.^22^, ^23^ We hypothesized that there are distinct developmental patterns of component-specific IgE responses to allergenic molecules from different sources, and that response patterns in early childhood may aid the prediction of clinical outcomes at a later date. To address our hypotheses, we used data from a well-characterized population-based birth cohort in which IgE responses to 112 allergen components were measured at 6 points from infancy to adolescence. We clustered allergen components based on component-specific IgE response profiles across subjects to identify cross-sectional sets of closely associated components at each age. We then determined the trajectories of these component clusters over time to investigate the evolution of sensitization patterns and examined their relationship with disease outcomes.

## Methods

### Study design, setting, and participants

The Manchester Asthma and Allergy Study (MAAS) is an unselected birth cohort; participants were recruited prenatally and followed prospectively.^24^, ^25^ The study was approved by the Research Ethics Committee; parents gave written informed consent. Participants attended review clinics at ages 1, 3, 5, 8, 11, and 16 years. Validated questionnaires were interviewer-administered to collect information on parentally reported symptoms, physician-diagnosed diseases, and treatments received. Blood samples were collected from participants who gave assent.

### Detection and annotation of component-specific IgE antibodies

We measured IgE to 112 components from 51 sources using ImmunoCAP ISAC (Thermo Fisher) at all 6 follow-ups. Levels of component-specific IgE antibodies were reported in ISAC standardized units. We discretized IgE data using a binary threshold (positive ≥0.30 ISAC standardized units).^17^ We used the following annotations for component-specific IgE antibody responses: active components—we considered components to be active if ≥3 participants had a positive IgE response at each time point^18^; and “drop-out” components—components that become inactive after having been active at an earlier time point.

### Definition of clinical outcomes at age 16 years

Current asthma is defined as any 2 of the following 3 features: (1) current wheeze (positive answer to the question “Has your child had wheezing or whistling in the chest in the last 12 months?”); (2) current use of asthma medication; (3) physician-diagnosed asthma ever.^26^

Current rhinitis is defined as a positive answer to the question “In the past 12 months, has your child had a problem with sneezing or a runny or blocked nose when he/she did not have a cold or the flu?”

### Statistical analysis

#### Statistical grouping of allergen components

For each time point, we analyzed the data for participants who had ≥1positive IgE component response and for the active allergen components^18^; we thus restricted our analysis to 10, 26, 63, 68, 71, and 72 active components at ages 1, 3, 5, 8, 11, and 16 years, respectively.

At each age, we inferred component clusters by clustering the data through Bayesian estimation of a mixture of Bernoulli distributions (Bernoulli mixture model). We inferred the model parameters, cluster membership, and number of clusters using an allocation sampler with an unknown number of mixture components (representing clusters in our terminology). This sampler is embedded in a Metropolis-coupled Markov chain Monte Carlo (MCMC) algorithm (details in this article's Online Repository at www.jacionline.org).^27^, ^28^, ^29^ The generated MCMC samples were postprocessed using the Equivalence Classes Representatives algorithm to overcome identifiability issues due to the label-switching problem.^30^, ^31^, ^32^, ^33^ The model, sampler, and the means to postprocess the results have been designed and implemented in R (http://www.r-project.org) by us and are published packages on CRAN (http://cran.r-project.org) available as bayesBinMix() and label.switching(), respectively.^34^, ^35^ Once the optimal number of clusters *K* was inferred at each age, the cluster membership was inferred conditional on that value.

#### Associations with clinical outcomes

CRD data from ages 1 and 3 years were sparse; we therefore evaluated the association between component clusters at ages 5 and 16 years with asthma and rhinitis at age 16 years. Children who did not respond to any active component were *a priori* assigned to a “nonsensitized” group. A child was classed as being sensitized to a component cluster if he/she responded to ≥1 component within the cluster. We examined the association between sensitization to component clusters and clinical outcomes (asthma, wheeze, and rhinitis) through logistic regression analyses (univariable and multiple); results are reported as odds ratios (OR) with 95% CIs.

## Results

### Participant flow and demographic data

Of 1184 children born into the cohort, CRD data were available for ≥1time point for 922 children. Participant flow is shown in Fig E1 in this article's Online Repository at www.jacionline.org. Number of children with CRD data at each follow-up, and the proportion with ≥1positive active component response are listed in Table E1 in this article's Online Repository at www.jacionline.org. Demographic and clinical characteristics are summarized in Table I; we observed some minor differences between children included and those excluded from this analysis, none of which were consistent across different ages.

**Table I:** Demographic characteristics of the study population at each time point and differences between children included and excluded from the analysis

Clinical variable	CRD data for ages					
	1 y	3 y	5 y	8 y	11 y	16 y
Overall						
Included	226/1184 (19.09)	248/1184 (20.95)	588/1184 (49.66)	543/1184 (45.86)	461/1184 (38.94)	361/1184 (30.49)
Excluded	958/1184 (80.91)	936/1184 (79.05)	596/1184 (50.34)	641/1884 (54.14)	723/1184 (61.06)	823/1184 (69.51)
Sex (male)						
Included	120/225 (53.33)	140/248 (56.45)	321/582 (55.15)	285/539 (52.88)	255/461 (55.31)	229/361 (63.43)
Excluded	522/959 (54.43)	502/936 (53.63)	321/602 (53.32)	357/645 (55.35)	387/723 (53.53)	413/823 (50.18)
*P* value	.82	.47	.57	.43	.59	**<.001**
Older siblings						
Included	114/204 (55.88)	136/239 (56.90)	332/572 (58.04)	304/539 (56.40)	254/458 (55.46)	191/359 (53.20)
Excluded	485/872 (55.62)	463/837 (55.32)	267/504 (52.98)	295/537 (54.93)	345/618 (55.83)	408/717 (56.90)
*P* value	1	.72	.11	.67	.95	.28
Maternal asthma						
Included	47/225 (20.89)	43/248 (17.34)	82/581 (14.11)	73/537 (13.59)	55/461 (11.93)	49/361 (13.57)
Excluded	125/954 (13.10)	129/931 (13.86)	90/598 (15.05)	99/642 (15.42)	117/718 (16.30)	123/818 (15.04)
*P* value	**.004**	.2	.71	.42	**.047**	.57
Maternal smoking (during pregnancy)						
Included	34/225 (15.11)	38/248 (15.32)	84/579 (14.51)	69/537 (12.85)	59/460 (12.83)	36/359 (10.03)
Excluded	140/952 (14.71)	136/929 (14.64)	90/598 (15.05)	105/640 (16.41)	115/717 (16.04)	138/818 (16.87)
*P* value	.96	.87	.86	.1	.15	**.003**
Paternal asthma						
Included	20/225 (8.89)	31/248 (12.50)	43/579 (7.43)	43/537 (8.01)	41/458 (8.95)	26/358 (7.26)
Excluded	65/951 (6.83)	54/928 (5.82)	42/597 (7.04)	42/639 (6.57)	44/718 (6.13)	59/818 (7.21)
*P* value	.35	**<.001**	.88	.4	.09	1
Maternal atopy						
Included	174/225 (77.33)	190/248 (76.61)	330/566 (58.30)	295/526 (56.08)	256/450 (56.89)	211/352 (59.94)
Excluded	508/921 (55.16)	492/898 (54.79)	352/580 (60.69)	387/620 (62.42)	426/696 (61.21)	471/794 (59.32)
*P* value	**<.001**	**<.001**	.45	**.034**	.16	.89
Paternal atopy						
Included	174/225 (77.33)	190/248 (76.61)	358/562 (63.70)	331/522 (63.41)	277/448 (61.83)	222/352 (63.07)
Excluded	542/912 (59.43)	526/889 (59.17)	358/575 (62.26)	385/615 (62.60)	439/689 (63.72)	494/785 (62.93)
*P* value	**<.001**	**<.001**	.66	.83	.56	1
Current asthma (age 16 y)						
Included	19/151 (12.58)	23/168 (13.69)	50/413 (12.11)	42/407 (10.32)	43/377 (11.41)	41/351 (11.68)
Excluded	71/585 (12.14)	67/568 (11.80)	40/323 (12.38)	48/329 (14.59)	47/359 (13.09)	49/385 (12.73)
*P* value	.99	.60	1.00	.10	.56	.75
Current wheeze (age 16 y)						
Included	25/149 (16.78)	31/167 (18.56)	71/413 (17.19)	62/405 (15.31)	66/382 (17.28)	55/354 (15.54)
Excluded	102/590 (17.29)	96/572 (16.78)	56/326 (17.18)	65/334 (19.46)	61/357 (17.09)	72/385 (18.70)
*P* value	.98	.67	1	.16	1	.30
Current rhinitis (age 16 y)						
Included	67/150 (44.67)	79/168 (47.02)	169/417 (40.53)	155/405 (38.27)	154/383 (40.21)	146/357 (40.90)
Excluded	242/594 (40.74)	230/576 (39.93)	140/327 (42.81)	154/339 (45.43)	155/361 (42.94)	163/387 (42.12)
*P* value	.44	.12	.58	.06	.50	.79
Asthma medication (age 16 y)						
Included	24/151 (15.89)	27/169 (15.98)	74/420 (17.62)	62/411 (15.09)	63/386 (16.32)	56/359 (15.60)
Excluded	104/600 (17.33)	101/582 (17.35)	54/331 (16.31)	66/340 (19.41)	65/365 (17.81)	72/392 (18.37)
*P* value	.76	.76	.71	.14	.66	.36
Asthma ever (age 16 y)						
Included	37/147 (25.17)	44/165 (26.67)	124/411 (30.17)	109/398 (27.39)	113/376 (30.05)	102/348 (29.31)
Excluded	183/584 (31.34)	176/566 (31.10)	96/320 (30.00)	111/333 (33.33)	107/355 (30.14)	118/383 (30.81)
*P* value	.18	.32	1	.10	1	.72
FEV_1_/FVC ratio (age 16 y)						
Included	88.41 (n = 131)	88.39 (n = 150)	88.04 (n = 372)	87.87 (n = 355)	88.16 (n = 356)	88.01 (n = 355)
Excluded	88.04 (n = 498)	88.03 (n = 479)	88.23 (n = 257)	88.43 (n = 274)	88.06 (n = 273)	88.25 (n = 274)
*P* value	.59	.59	.75	.77	.51	0.44
Sensitization (SPT)						
Included	23/222 (10.36)	64/245 (26.12)	177/572 (30.94)	155/531 (29.19)	164/455 (36.04)	188/341 (55.13)
Excluded	33/282 (11.70)	161/738 (21.82)	117/391 (29.92)	159/396 (40.15)	116/340 (34.12)	134/259 (51.74)
*P* value	.74	.19	.79	**<.001**	.63	.46

*FVC*, forced vital capacity.

Values are n/n (%) unless otherwise indicated. Boldface values are statistically significant.

### Component-specific IgE responses across childhood

#### Active, inactive, and components that dropped out

A total of 86 components were active for ≥1time point. Components that were inactive at all ages (n = 26) are listed in Table E2 in this article's Online Repository at www.jacionline.org; note, 1 or 2 children had positive IgE to some of these components, and for 3 components (Asp f 1, Bla g 5, Hev b 5), there was no positive response in any subject at any age. Inactive components at each age are listed in Table E3 in this article's Online Repository at www.jacionline.org.

Table E4 in this article's Online Repository at www.jacionline.org shows 24 components that dropped out (not necessarily permanently) and the number of children who were sensitized to these components. Fig E2A, Fig E2B in this article's Online Repository at www.jacionline.org shows detailed longitudinal response profiles of each component that ever became inactive after first becoming active, for each child who has ever responded; for 12 components, we linked their drop-out to the resolution of sensitization (Fig E2, *A*), and for the remaining 12 to the absence at subsequent follow-up of previously sensitized subjects (Fig E2, *B*).

### Component clusters at each time point and their longitudinal flows

Table II shows the number of component clusters inferred at each time point, and their posterior probabilities determined using Bayesian inference. The optimal solution identified 1 sensitization cluster at age 1, 3 at age 3, 4 at ages 5 and 8, 5 at age 11, and 6 at age 16 years. The posterior probabilities for the most probable number of clusters were at least 0.87 for the first 5 time points and remained above 0.70 at age 16 years. Tables E5 to E10 in this article's Online Repository at www.jacionline.org list components in each cluster at each time point.

**Table II:** Inference of the number of component clusters at each time point

Age (y)	*K*_max_	*P*(*K*), where *K* =							
		1	2	3	4	5	6	7	8
1	9	**.8958**	.0932	.0106	.0004	0	0	0	0
3	25	.0004	.0208	**.8784**	.0942	.0062	0	0	0
5	25	.0004	0	.0012	**.9548**	.0426	.0010	0	0
8	25	.0004	0	0	**.9440**	.0532	.0024	0	0
11	25	0	.0004	.0012	.0032	**.9416**	.0516	.0012	.0008
16	25	0	.0004	0	.0000	.2536	**.7066**	.0358	.0036

The posterior probability of the number of clusters *K* was determined through Bayesian inference with a Bernoulli mixture model applied to binarized sensitization data from all subjects. The most probable *K* for each time point is highlighted in boldface.

**Fig 1:**
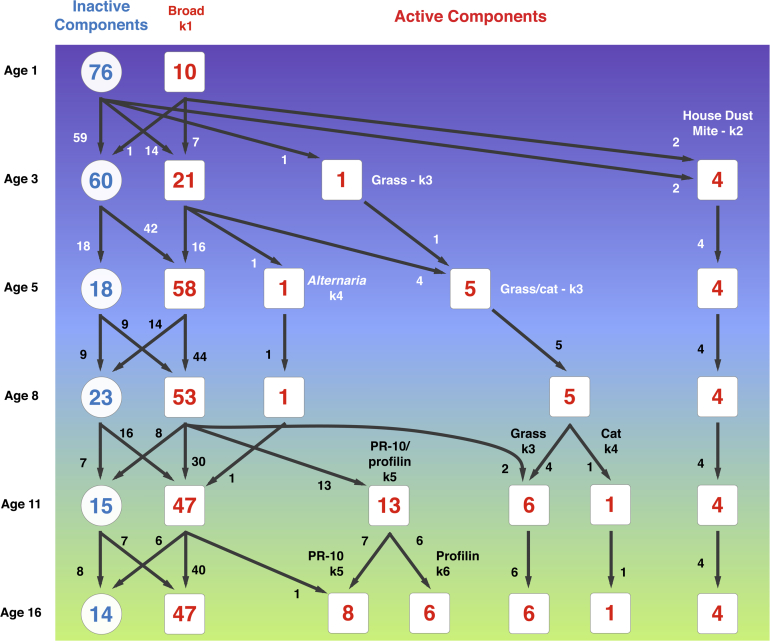
Clustering active IgE components throughout childhood. Cluster membership was determined using a Bernoulli mixture model applied to binarized sensitization data from all subjects.

We qualitatively labelled clusters at each age based on the profile of allergen components to which sensitization occurred. Fig 1 shows the number of active components contained within each cluster for each time point (red), how many components were inactive (blue), and how many components were shared between clusters at adjacent time points.

The “broad” cluster comprising components originating from multiple sources was the only cluster identified at every time point. Components forming this cluster differed at different ages; Table E11 in this article's Online Repository at www.jacionline.org shows 24 components that were only ever assigned to the broad cluster.

From age 3 onward, the “HDM” cluster formed and remained unchanged by age 16 years; it consists of 4 mite components (Der p 1-2, Der f 1-2). Also at age 3 years, the “grass” cluster emerged, consisting of a single component (Phl p 1) (Table E6). This cluster absorbed an additional 3 grass components, as well as cat component Fel d 1 to form the “grass/cat” cluster at age 5 years (Table E7). The membership of this cluster remained unchanged at age 8 years, although Fel d 1 assignment probability was reduced from >0.95 at age 5 to 0.70 (Table E8). A further cluster that was shared across ages 5 and 8 years was the “*Alternaria*” cluster, comprising only Alt a 1. At age 11 years, this component was reabsorbed by the broad cluster, the only component to do so throughout this flow (Fig 1).

Two new clusters formed at age 11 years: the “cat” cluster (comprising Fel d 1) and the “PR-10/profilin” cluster (Table E9). The latter was composed solely of components that have moved from the broad cluster at age 8 years. Additional grass components were absorbed from the broad into the grass cluster at age 11 years (Phl p 2 and Phl p 6). This cluster divided at age 16 years into 2: “PR-10” and “profilin” (Table E10); other clusters remained unchanged at age 16 years.

**Fig 2:**
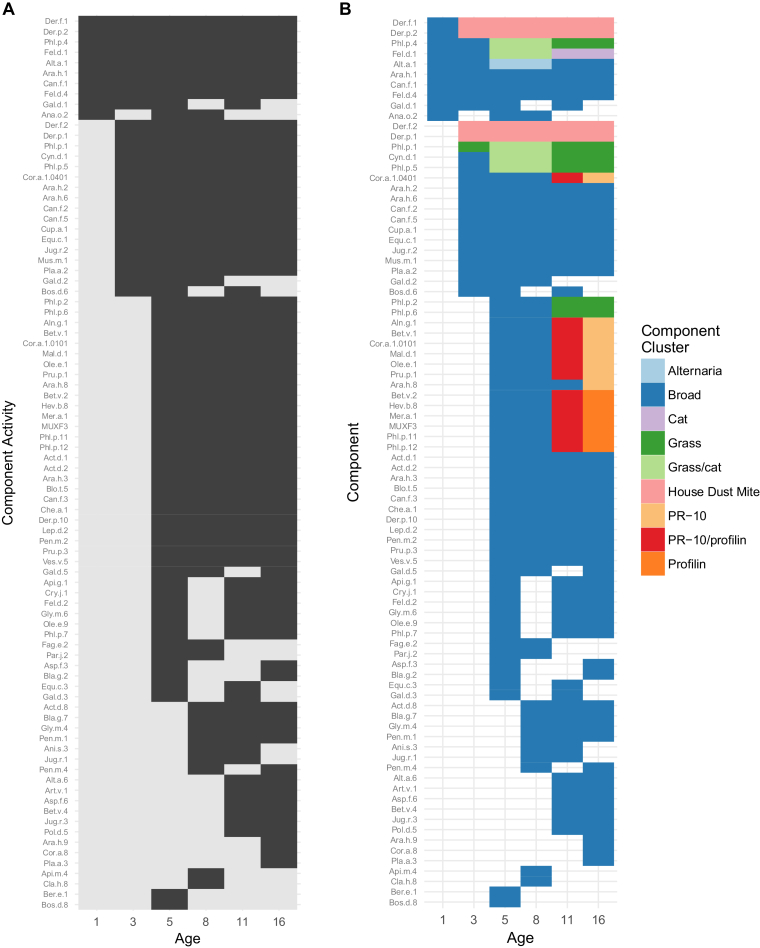
The change of activity across all components, and their cluster membership during childhood. **A,** Individual allergen component activity at each age; *black* for active, *gray* for inactive. **B,** Color-coded by cluster membership; *blank* if inactive at a time point. Allergen components are sorted according to the time point of first activity, then by total number of time points active at, then by cluster membership, and finally based on persistence (ie, do the components remain active after first becoming so). Exceptions are the components that are active at only 1 time point, which appear at the bottom.

Fig 2 shows the change of activity across all components, and their cluster membership during childhood. Not all 86 components that were ever active across the 6 time points were active at every point. The inactive components populate the nodes in the left-hand pathway of Fig 1. All 24 components that dropped-out (Table E4) were assigned only to the broad cluster. Components from all other clusters remained active once they first became so.

### Sensitization to component clusters and clinical outcomes

The frequencies of component cluster sensitization profiles at ages 5 and 16 are shown in Table E12 in this article's Online Repository at www.jacionline.org. For children who were sensitized to ≥1 cluster at age 5 years, the most common response (n = 42) was to the grass cluster only. The confusion matrix in Table E13 in this article's Online Repository at www.jacionline.org displays the number of children who shared sensitization to the clusters at ages 5 and 16 years, for 255 children who had CRD data at both follow-ups. Of 62 children who were sensitized to the broad cluster at age 5 years, 53 went on to respond to the grass cluster at age 16 years, with 51 remaining sensitized to the broad cluster as well.

#### Univariable analyses

Sensitization to any of the component clusters at ages 5 and 16 years was associated with a significantly higher risk of asthma, wheeze, and rhinitis at age 16 (Fig E3A, Fig E3B in this article's Online Repository at www.jacionline.org). However, the associations differed at different ages. At age 16 years, we observed the highest risk of asthma in relation to contemporaneous sensitization to the HDM cluster (OR: 12.4; 95% CI: 4.2-36.8; *P* < .001) (Fig E3, *A*), but the strongest associate of asthma in adolescence in relation to sensitization at age 5 years was conferred by sensitization to the grass/cat cluster (OR: 10.0; 95% CI: 4.6-21.7; *P* < .001) (Fig E3, *B*). Similarly, the risk of rhinitis was greatest for those sensitized to the profilin cluster at age 16 years (OR: 30.6; 95% CI: 14.9-62.9; *P* < .001), but at age 5 years, the strongest associate of subsequent rhinitis was sensitization to the broad cluster (OR: 7.0; 95% CI: 2.9-11.4; *P* < .001).

#### Multiple logistic regression

**Fig 3:**
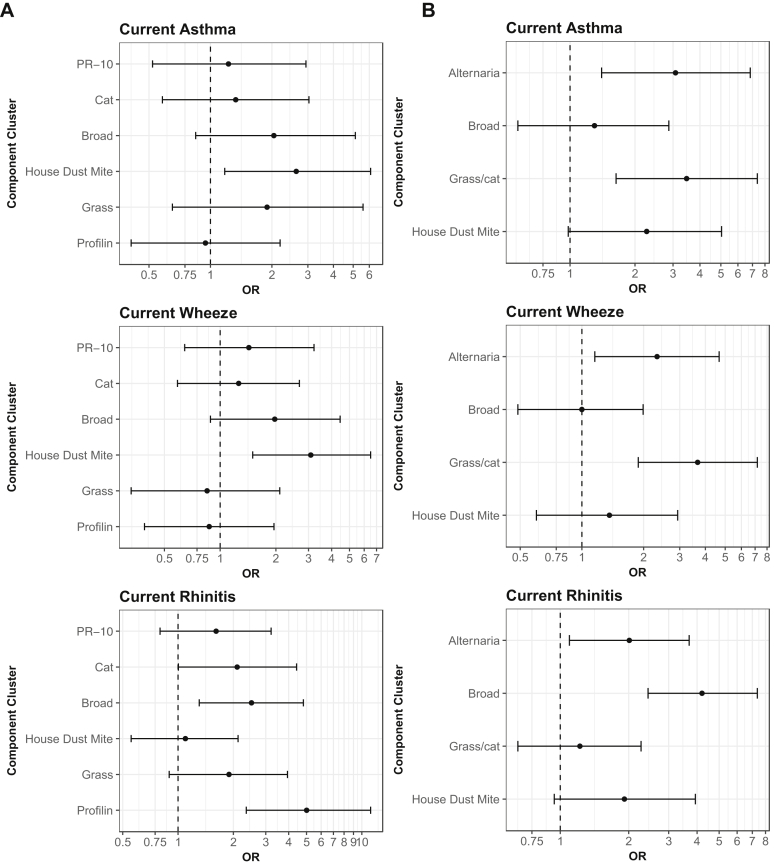
ORs and 95% CIs from multiple logistic regression, for asthma and rhinitis at age 16 years based on subjects' reduced responses to **(A)** component clusters at age 16 and **(B)** component clusters at age 5 years.

In the analysis that evaluated the association between sensitization to component clusters at age 16 years and contemporaneous allergic diseases (Fig 3, *A*), only sensitization to the HDM cluster was associated with the increased risk of asthma and wheeze (OR: 2.6; 95% CI: 1.2-6.1; *P* < .05, and OR: 3.1; 95% CI: 1.5-6.5; *P* < .01, respectively). When we extended the time frame to investigate the relationship between cluster sensitization at age 5 years and clinical outcomes at age 16 (Fig 3, *B*, and Table E14 in this article's Online Repository at www.jacionline.org), there was no significant association between asthma and sensitization to the broad and HDM clusters, and the strongest risk of subsequent asthma was conferred by sensitization to the grass/cat and *Alternaria* clusters (OR: 3.5; 95% CI: 1.6-7.4; *P* < .01, and OR: 3.1; 95% CI: 1.4-6.8; *P* = .005, respectively). Similarly, the magnitude of risk for contemporaneous rhinitis was greatest among children sensitized to the profilin cluster (OR: 5.0; 95% CI: 2.3-11.2; *P* < .001), but at age 5 years, the strongest predictor of subsequent rhinitis was sensitization to the broad cluster (OR: 4.2; 95% CI: 2.4-7.4; *P* < .001).

## Discussion

We describe the architecture of the evolution of IgE responses to multiple allergen components throughout childhood, taking into account responses to >100 allergenic molecules. By applying novel machine learning techniques to CRD sensitization data from infancy to adolescence among children from a population-based birth cohort, we identified latent structure in the diversification of the IgE responses during childhood (Figs 1 and 2). Our comprehensive description of the patterns of IgE responses to multiple components from infancy to adolescence demonstrated that the timing of onset of specific patterns of sensitization may be among the important indicators of the subsequent risk of allergic disease (Fig 3). While children were frequently sensitized to ≥1cluster, sensitization to distinct clusters was associated with different clinical presentations, indicating that some sensitization patterns pose greater risk for the development of specific clinical symptoms than others do.

One of the limitations of our study includes the lack of potentially important components that are not included on the ISAC chip, such as those from HDM and fungi (eg, ISAC has 6 of 109 fungal allergens identified by the International Union of Immunological Societies). This may be among the reasons why the *Alternaria* cluster contained only 1 component (Alt a 1). Of note, sensitization to this small cluster at age 5 years conferred a strong risk for asthma in later life. This is also of relevance to the HDM cluster, which was the only cluster to remain unchanged once it had formed at age 3, with Der p 1 being the dominant component. A recent study that measured IgE response to a broader range of HDM allergens has shown that sensitization increases in breadth with respect to the number of recognized allergenic molecules during the first decade of life.^20^ It is possible that we would have observed similar “epitope spreading” if we measured IgE to a greater number of HDM allergens.

We acknowledge that the number of sensitized children in early life was small (only 10 of 226 at age 1 year), and we cannot exclude the possibility that this may have introduced bias in our analyses. However, we believe that presenting data at all ages is important to ascertain the life-course perspective.

We were unable to determine the effect of partial or complete sensitization to each cluster and the relative importance of sensitization to specific “lead” component(s) compared with the number of components within each cluster. This question will need to be addressed in future studies. We also acknowledge that our study population comes from a specific geographical area, and that different component clusters may arise in areas with different patterns of allergen exposure, or by using a more comprehensive allergen panel. Thus, different components may be informative in a different geographical or analytical context.

Allergen-specific IgG may be important in modulating the consequences of T_h_2 immunity in IgE-sensitized children.^36^, ^37^ However, exploring IgG responses and IgG/IgE ratios was beyond the scope of the current study.

Our method identified cross-sectional sensitization patterns and their longitudinal trajectories. It is of note that despite the increasing number of active components, the varying number of participants, and the derivation of our clusters being independent at different time points, the components allocated to clusters were strikingly consistent across time, and the assignment probabilities were very high. Our finding that IgE reactivity diversifies in molecular heterogeneity, and that component-specific IgE responses are assigned to a steadily diversifying set of clusters, is consistent with the “molecular spreading” hypothesis,^38^ and indirectly supports our findings, which suggested the existence of multiple subtypes of allergic sensitization.^39^, ^40^ The increasing number of component-specific IgEs to which individual patients are responding in later childhood (polysensitization) is associated with increasing severity of allergic disease,^18^ but may also indicate that the sensitization process has started earlier. Our data extend the relatively broad concepts of “polysensitization” and “early sensitization” to demonstrate that for a more precise ascertainment of future and current risk of allergic diseases, we need accurate information about the specific patterns of sensitization to unique sets of allergenic molecules, as well as the timing of onset of sensitization.

Our results suggest that the timing of onset of specific sensitization patterns may be a key indicator of future risk, and that apparently similar cross-sectional profiles of component-specific IgE responses may have different clinical associations depending on the age at which they emerge. This expands on our previous study^19^ in which we used a limited number of timothy grass and HDM components, which described 2 grass pollen IgE trajectories (“late onset” and “early onset”). Although the progression of IgE component responses over time was identical in the 2 trajectories, following the sequence of Phl p 1/5→Phl p 2/4/6→Phl p 7/11/12, their clinical associations were different. The early onset trajectory (in which Phl p 1/5 IgE responses emerged in preschool age) was associated with asthma and multimorbidity, while the late onset trajectory (in which the same component-specific IgE responses were first observed in school-age children) was associated with rhinitis.^19^ At the time when we conducted previous analyses, limitations including computing power and available methodologies precluded us from investigating the developmental pathways across all 112 components. In the current study, a more complex structure emerged. This is highlighted by the emergence of grass/cat cluster at age 5 years, in which allergenic proteins from diverse sources, and with a fundamentally different function, clustered together. Although it may appear counterintuitive that Fel d 1 should be in the same cluster as the timothy and Bermuda grass components, the assignment probability for the cat component belonging to this cluster was very high (0.97). The response to this cluster was strongly associated with asthma at age 16 years (3.5-fold increase in risk). This may suggest that the latent structure of IgE component clusters is not only a reflection of the source of allergens, or the function of allergenic molecules (as suggested by 1 of our previous studies),^18^ but that it may also be a marker of the underlying pathophysiological processes leading to the development of distinct clinical phenotypes. Thus, a possible reason why cat and grass components clustered together in 5-year-old children from our area may be due to the IgE responses to these components foreshadowing the pathophysiological pathway leading to asthma (although we acknowledge that these IgE responses do not necessarily have to be causal).

In conclusion, different patterns of IgE responses to multiple allergen components evolve throughout childhood and can be uncovered using machine learning. Specific sensitization patterns in early childhood are predictive of distinct allergic phenotypes in adolescence. Better resolution of longitudinal patterns may contribute to a better understanding of the pathophysiological processes giving rise to different allergic diseases and may facilitate the development of diagnostic algorithms, which can be used for the prediction of current and future risk.

**Clinical implications**

Development of different clinical phenotypes of allergic diseases may be predicted by the distinct patterns of IgE responses to multiple allergenic proteins.

## Supplementary data

Online Repository text

Tables E1-E14

Fig E1

Fig E2A

Fig E2B

Fig E3A

Fig E3B
