# Supplementary material for: Evolution of IgE responses to multiple allergen components throughout childhood
Source: J Allergy Clin Immunol. 2018 Oct;142(4):1322–30. doi: 10.1016/j.jaci.2017.11.064 (PMC6170973; doi:10.1016/j.jaci.2017.11.064)
Supplement: Online Repository text [file mmc1.docx]

**Evolution of IgE responses to multiple allergen components throughout childhood**

Rebecca Howard MSc^1^, Danielle Belgrave PhD^2^, Panagiotis Papastamoulis PhD^1^, Angela Simpson MD PhD^3^, Magnus Rattray PhD^1^, Adnan Custovic MD PhD^2^

1. Division of Informatics, Imaging and Data Sciences, Faculty of Biology, Medicine and Health, University of Manchester, Manchester, UK.

2. Section of Paediatrics, Department of Medicine, Imperial College London, UK.

3. Division of Infection, Immunity and Respiratory Medicine, University of Manchester and University Hospital of South Manchester, Manchester Academic Health Sciences Centre, Manchester, United Kingdom.

**ONLINE DATA SUPPLEMENT**

**METHODS**

***Screening & Recruitment***

All pregnant women were screened for eligibility at antenatal visits (8^th^-10^th^ week of pregnancy). Of the 1499 couples who met the inclusion criteria (<10 weeks of pregnancy, maternal age >18 years), 288 declined to take part and 27 were lost to follow-up between recruitment and birth of a child. A total of 1184 participants had some evaluable data.

***Follow-up***

Children have been followed prospectively, and attended review clinics at ages 1, 3, 5, 8, 11 and 16 years. At age 1 year, only children with either both atopic parents, or no atopic parents were invited to attend for clinical follow up. At all other time points for all other measures all children were invited to participate.

***Statistical grouping of allergen components***

We assumed that there exist clusters of components to which subjects have similar IgE responses (i.e., either being sensitized or not to most of the components within the same cluster). At each age, we inferred component clusters by clustering the data through Bayesian estimation of a mixture of Bernoulli distributions (Bernoulli Mixture Model–BMM). The BMM method provides a fully Bayesian method which can effectively deal with missing data and an unknown number of clusters. This method was shown to achieve a much better estimation of the number of clusters compared to the EM implementation contained within the FlexMix() R package (for detailed description of the methodology and benchmarking, please see Panagiotis Papastamoulis and Magnus Rattray , The R Journal (2017) 9:1, pages 403-420.; (URL: <https://journal.r-project.org/archive/2017/RJ-2017-022/index.html>). Specifically, once the total number of clusters exceeds 4, our Bayesian method does not underestimate the correct number of clusters, unlike the EM implementation.

The observed likelihood for a binary data matrix ***x*** under the BMM model is given by:

$$L_{K}\left( \boldsymbol{p, \theta};\boldsymbol{x} \right)= \prod_{i=1}^{n} \sum_{k=1}^{K} p_{k}\prod_{j=1}^{d} {\theta_{kj}^{x_{ij}}\left( 1-\theta_{kj} \right)}^{1-x_{ij}}$$

where $\theta_{jk}$ is between 0 and 1 and represents the frequency of sensitisation to component *j* for subjects in cluster *k* and $p_{k}$ represents the weight of cluster *k* which is the prior probability that a subject belongs to that cluster.

***Associations with clinical outcomes***

The relationships between a subject’s responses to the BMM’s cluster output and their disease outcomes were assessed using univariable and multiple logistic regression analyses (adjusting for sensitization to each of the component clusters, and the sex of the child). In addition to frequentist intervals, we calculated Bayesian posterior credible regions.

**RESULTS**

**Table S1**. Number of children with component-resolved diagnostics data and proportion of those with at least one positive allergen component response at each follow-up

**Table S2.** The list of 26 components which were labelled inactive at all 6 time points

**Table S3.** Components labelled inactive for ages a) 1, b) 3, c) 5, d) 8, e) 11, and f) 16 years.

**Table S4.** Subject response totals for each of the allergen components which “dropped-out” (i.e. become inactive after first being active). Italics indicates when that component is “Active”; bold if “inactive” but 1 or 2 subjects have positively responded to that component at that time point; otherwise if “inactive” and no subjects have positively responded at that time.

**Table S5.** Age 1’s component cluster members and the number of children that respond to each member.

**Table S6.** Age 3’s component cluster members, number of children that respond to each member, and the assignment probability to the most probable cluster.

**Table S7:** Age 5’s component cluster members, number of children that respond to each member, and the assignment probability to the most probable cluster.

**Table S8:** Age 8’s component cluster members, number of children that respond to each member, and the assignment probability to the most probable cluster.

**Table S9:** Age 11’s component cluster members, number of children that respond to each member, and the assignment probability to the most probable cluster.

**Table S10:** Age 16’s component cluster members, number of children that respond to each member, and the assignment probability to the most probable cluster; Age 16, K = 6

**Table S11**. Components which were only ever assigned to the “Broad” cluster.

**Table S12.** Frequencies for each subject’s reduced response to the component clusters found at **(a)** age 5, and **(b)** age 16.

**Table S13.** Confusion matrix for the reduced response frequencies of the 255 children that had ISAC data for both ages 5 and 16. The clusters from age 5: the left, the clusters from age 16: the top. Note that rows and columns do not sum to the totals, as responses to the clusters are not mutually exclusive. Note the relatively small proportion of children that have reduced responses to each of the clusters at each of these ages (but particularly at age 5), acting as a main source for a wide range in confidence intervals for associations with clinical outcomes.

**Table S14.** C-statistic reported for each of the multivariate logistic regression models applied to both age 5 and age 16’s cluster response data, with relation to rhinitis and asthma-related clinical outcomes at age 16.

**Figure S1.** CONSORT diagram for participant flow

**Figure S2**. Response profiles for each component that ever becomes inactive after first becoming active, for each child that ever responds to each of these components. Darker blue fill for when the component is active at that time point i.e. at least three subjects had a positive response to that component at that time point. Points represent when ISAC data is available for that child. Response: positive (red) or negative (blue).

1. Components whose drop-out can be ascribed to desensitisation *i.e.* if all children who were positive to a component at the time point preceding the drop-out were still positive.
2. The remaining 12 components, all of whose drop-outs can be explained by the subject loss to follow-up.

**Figure S3.** Odds ratios and 95% CIs from univariable logistic regression for asthma and rhinitis at age 16, based on subjects’ reduced responses to component clusters at **(a)** age 16; **(b)** age 5. Bayesian posterior credible regions were also computed, and agreed closely with the 95% CIs shown.

b)
