# Supplementary material for: Evolution of IgE responses to multiple allergen components throughout childhood
Source: J Allergy Clin Immunol. 2018 Oct;142(4):1322–30. doi: 10.1016/j.jaci.2017.11.064 (PMC6170973; doi:10.1016/j.jaci.2017.11.064)
Supplement: Tables E1-E14 [file mmc2.doc]

Table S1

|  |  |  |  |  |  |  |  |
| --- | --- | --- | --- | --- | --- | --- | --- |
|  |  |  |  |  |  |  |  |
|  | **Age** |  | **Number of** |  | **Children responding to** | |  |
|  | **(y)** |  | **children tested** |  | **≥1 active components (%)** | |  |
|  |  |  |  |  |  |  |  |
|  |  |  |  |  |  |  |  |
|  | 1 |  | 226 |  | 43 | .(19.0%) |  |
|  | 3 |  | 248 |  | 88 | .(35.5%) |  |
|  | 5 |  | 588 |  | 253 | .(43.0%) |  |
|  | 8 |  | 543 |  | 256 | .(47.1%) |  |
|  | 11 |  | 461 |  | 220 | .(47.7%) |  |
|  | 16 |  | 361 |  | 207 | .(57.3%) |  |
|  |  |  |  |  |  |  |  |
|  |  |  |  |  |  |  |  |

Table S2

|  |  |  |
| --- | --- | --- |
|  |  |  |
|  | **Perennially inactive** |  |
| **components** | | |
|  |  |  |
|  |  |  |
|  | Act.d.5 |  |
|  | Amb.a.1 |  |
|  | Ani.s.1 |  |
|  | Api.m.1 |  |
|  | Art.v.3 |  |
|  | Asp.f.1 |  |
|  | Bla.g.1 |  |
|  | Bla.g.5 |  |
|  | Bos.d.4 |  |
|  | Bos.d.5 |  |
|  | Bos.d.lactoferrin |  |
|  | Cor.a.9 |  |
|  | Gad.c.1 |  |
|  | Gly.m.5 |  |
|  | Hev.b.1 |  |
|  | Hev.b.3 |  |
|  | Hev.b.5 |  |
|  | Hev.b.6.01 |  |
|  | Ole.e.7 |  |
|  | Pla.a.1 |  |
|  | Pla.l.1 |  |
|  | Sal.k.1 |  |
|  | Ses.i.1 |  |
|  | Tri.a.14 |  |
|  | Tri.a.19.0101 |  |
|  | Tri.a.aA_TI |  |
|  |  |  |
|  |  |  |

Table S3a

|  |  |  |  |  |  |
| --- | --- | --- | --- | --- | --- |
|  |  |  |  |  |  |
| **Age 1** | | | | |  |
|  |  |  |  |  |  |
|  |  |  |  |  |  |
| **No children** | | | **1 child** | **2 children** |  |
| **positive** | | | **positive** | **positive** |  |
|  |  |  |  |  |  |
|  |  |  |  |  |  |
| Act.d.1 | Bos.d.8 | Mer.a.1 | Ara.h.6 | Ara.h.2 |  |
| Act.d.2 | Bos.d.lactoferrin | Mus.m.1 | Bos.d.6 | Bla.g.7 |  |
| Act.d.5 | Can.f.3 | MUXF3 | Can.f.2 | Der.f.2 |  |
| Act.d.8 | Can.f.5 | Ole.e.1 | Cor.a.1.0401 | Der.p.1 |  |
| Aln.g.1 | Che.a.1 | Ole.e.7 | Gal.d.3 | Gal.d.2 |  |
| Alt.a.6 | Cla.h.8 | Ole.e.9 | Gly.m.5 | Gly.m.6 |  |
| Amb.a.1 | Cor.a.1.0101 | Par.j.2 | Pen.m.1 |  |  |
| Ani.s.1 | Cor.a.8 | Pen.m.2 |  |  |  |
| Ani.s.3 | Cor.a.9 | Pen.m.4 |  |  |  |
| Api.g.1 | Cry.j.1 | Phl.p.1 |  |  |  |
| Api.m.1 | Cup.a.1 | Phl.p.11 |  |  |  |
| Api.m.4 | Cyn.d.1 | Phl.p.12 |  |  |  |
| Ara.h.3 | Der.p.10 | Phl.p.2 |  |  |  |
| Ara.h.8 | Equ.c.1 | Phl.p.5 |  |  |  |
| Ara.h.9 | Equ.c.3 | Phl.p.6 |  |  |  |
| Art.v.1 | Fag.e.2 | Phl.p.7 |  |  |  |
| Art.v.3 | Fel.d.2 | Pla.a.1 |  |  |  |
| Asp.f.1 | Gad.c.1 | Pla.a.2 |  |  |  |
| Asp.f.3 | Gal.d.5 | Pla.a.3 |  |  |  |
| Asp.f.6 | Gly.m.4 | Pla.l.1 |  |  |  |
| Ber.e.1 | Hev.b.1 | Pol.d.5 |  |  |  |
| Bet.v.1 | Hev.b.3 | Pru.p.1 |  |  |  |
| Bet.v.2 | Hev.b.5 | Pru.p.3 |  |  |  |
| Bet.v.4 | Hev.b.6.01 | Sal.k.1 |  |  |  |
| Bla.g.1 | Hev.b.8 | Ses.i.1 |  |  |  |
| Bla.g.2 | Jug.r.1 | Tri.a.14 |  |  |  |
| Bla.g.5 | Jug.r.2 | Tri.a.19.0101 |  |  |  |
| Blo.t.5 | Jug.r.3 | Tri.a.aA_TI |  |  |  |
| Bos.d.4 | Lep.d.2 | Ves.v.5 |  |  |  |
| Bos.d.5 | Mal.d.1 |  |  |  |  |
|  |  |  |  |  |  |
|  |  |  |  |  |  |

Table S3b

|  |  |  |  |  |
| --- | --- | --- | --- | --- |
|  |  |  |  |  |
| **Age 3** | | | |  |
|  |  |  |  |  |
|  |  |  |  |  |
| **No children** | | **1 child** | **2 children** |  |
| **positive** | | **positive** | **positive** |  |
|  |  |  |  |  |
|  |  |  |  |  |
| Act.d.2 | Der.p.10 | Act.d.1 | Bet.v.1 |  |
| Act.d.5 | Gad.c.1 | Aln.g.1 | Bos.d.lactoferrin |  |
| Act.d.8 | Gal.d.5 | Ana.o.2 | Can.f.3 |  |
| Alt.a.6 | Gly.m.4 | Ani.s.1 | Cor.a.9 |  |
| Amb.a.1 | Hev.b.1 | Ara.h.3 | Cry.j.1 |  |
| Ani.s.3 | Hev.b.3 | Bla.g.2 | Gal.d.3 |  |
| Api.g.1 | Hev.b.5 | Bos.d.4 | Hev.b.8 |  |
| Api.m.1 | Hev.b.6.01 | Bos.d.5 | MUXF3 |  |
| Api.m.4 | Jug.r.3 | Bos.d.8 | Phl.p.11 |  |
| Ara.h.8 | Lep.d.2 | Equ.c.3 | Phl.p.6 |  |
| Ara.h.9 | Mer.a.1 | Fag.e.2 | Pol.d.5 |  |
| Art.v.1 | Ole.e.1 | Fel.d.2 |  |  |
| Art.v.3 | Ole.e.7 | Gly.m.5 |  |  |
| Asp.f.1 | Par.j.2 | Gly.m.6 |  |  |
| Asp.f.3 | Pen.m.1 | Jug.r.1 |  |  |
| Asp.f.6 | Phl.p.12 | Mal.d.1 |  |  |
| Ber.e.1 | Phl.p.7 | Ole.e.9 |  |  |
| Bet.v.2 | Pla.a.1 | Pen.m.2 |  |  |
| Bet.v.4 | Pla.a.3 | Pen.m.4 |  |  |
| Bla.g.1 | Pla.l.1 | Phl.p.2 |  |  |
| Bla.g.5 | Pru.p.3 | Pru.p.1 |  |  |
| Bla.g.7 | Sal.k.1 |  |  |  |
| Blo.t.5 | Ses.i.1 |  |  |  |
| Che.a.1 | Tri.a.14 |  |  |  |
| Cla.h.8 | Tri.a.19.0101 |  |  |  |
| Cor.a.1.0101 | Tri.a.aA_TI |  |  |  |
| Cor.a.8 | Ves.v.5 |  |  |  |
|  |  |  |  |  |
|  |  |  |  |  |

Table S3c

|  |  |  |
| --- | --- | --- |
|  |  |  |
| **Age 5** | | |
|  |  |  |
|  |  |  |
| **No children** | **1 child** | **2 children** |
| **positive** | **positive** | **positive** |
|  |  |  |
|  |  |  |
| Act.d.5 | Act.d.8 | Ara.h.9 |
| Alt.a.6 | Ani.s.1 | Bet.v.4 |
| Amb.a.1 | Ani.s.3 | Bos.d.5 |
| Api.m.1 | Api.m.4 | Gad.c.1 |
| Art.v.1 | Art.v.3 | Gly.m.5 |
| Asp.f.1 | Asp.f.6 | Jug.r.1 |
| Bla.g.1 | Bos.d.4 | Pen.m.1 |
| Bla.g.5 | Bos.d.lactoferrin | Pen.m.4 |
| Bla.g.7 | Cla.h.8 | Pol.d.5 |
| Hev.b.1 | Cor.a.8 |  |
| Hev.b.3 | Cor.a.9 |  |
| Hev.b.5 | Gly.m.4 |  |
| Hev.b.6.01 | Jug.r.3 |  |
| Ole.e.7 | Sal.k.1 |  |
| Pla.a.1 | Tri.a.aA_TI |  |
| Pla.a.3 |  |  |
| Pla.l.1 |  |  |
| Ses.i.1 |  |  |
| Tri.a.14 |  |  |
| Tri.a.19.0101 |  |  |
|  |  |  |
|  |  |  |

Table S3d

|  |  |  |
| --- | --- | --- |
|  |  |  |
| **Age 8** | | |
|  |  |  |
|  |  |  |
| **No children** | **1 child** | **2 children** |
| **positive** | **positive** | **positive** |
|  |  |  |
|  |  |  |
| Act.d.5 | Amb.a.1 | Alt.a.6 |
| Ani.s.1 | Art.v.1 | Api.g.1 |
| Api.m.1 | Ber.e.1 | Ara.h.9 |
| Art.v.3 | Bla.g.1 | Asp.f.3 |
| Asp.f.1 | Bos.d.4 | Asp.f.6 |
| Bla.g.5 | Bos.d.6 | Bet.v.4 |
| Bos.d.5 | Bos.d.8 | Bla.g.2 |
| Bos.d.lactoferrin | Cry.j.1 | Cor.a.9 |
| Cor.a.8 | Equ.c.3 | Gal.d.1 |
| Gly.m.5 | Fel.d.2 | Gly.m.6 |
| Hev.b.1 | Gad.c.1 | Ole.e.9 |
| Hev.b.3 | Gal.d.3 | Phl.p.7 |
| Hev.b.5 | Jug.r.3 | Sal.k.1 |
| Hev.b.6.01 | Pla.a.1 |  |
| Ole.e.7 | Pla.a.3 |  |
| Ses.i.1 | Pla.l.1 |  |
| Tri.a.19.0101 | Pol.d.5 |  |
| Tri.a.aA_TI | Tri.a.14 |  |
|  |  |  |
|  |  |  |

Table S3e

|  |  |  |
| --- | --- | --- |
|  |  |  |
| **Age 11** | | |
|  |  |  |
|  |  |  |
| **No children** | **1 child** | **2 children** |
| **positive** | **positive** | **positive** |
|  |  |  |
|  |  |  |
| Act.d.5 | Ani.s.1 | Ara.h.9 |
| Amb.a.1 | Asp.f.3 | Art.v.3 |
| Ana.o.2 | Ber.e.1 | Bos.d.4 |
| Api.m.1 | Bla.g.1 | Bos.d.5 |
| Api.m.4 | Bos.d.lactoferrin | Bos.d.8 |
| Asp.f.1 | Cor.a.8 | Gal.d.2 |
| Bla.g.2 | Cor.a.9 | Gly.m.5 |
| Bla.g.5 | Gad.c.1 | Hev.b.3 |
| Cla.h.8 | Hev.b.1 | Hev.b.6.01 |
| Fag.e.2 | Ole.e.7 | Pla.a.1 |
| Gal.d.5 | Par.j.2 | Pla.a.3 |
| Hev.b.5 | Pen.m.4 | Pla.l.1 |
| Sal.k.1 | Ses.i.1 | Tri.a.19.0101 |
| Tri.a.14 | Tri.a.aA_TI |  |
|  |  |  |
|  |  |  |

Table S3f

|  |  |  |
| --- | --- | --- |
|  |  |  |
| **Age 16** | | |
|  |  |  |
|  |  |  |
| **No children** | **1 child** | **2 children** |
| **positive** | **positive** | **positive** |
|  |  |  |
|  |  |  |
| Amb.a.1 | Act.d.5 | Ani.s.3 |
| Ani.s.1 | Ana.o.2 | Api.m.4 |
| Asp.f.1 | Api.m.1 | Bos.d.4 |
| Bla.g.5 | Art.v.3 | Bos.d.6 |
| Bos.d.8 | Ber.e.1 | Cla.h.8 |
| Fag.e.2 | Bla.g.1 | Cor.a.9 |
| Hev.b.1 | Bos.d.5 | Equ.c.3 |
| Hev.b.3 | Bos.d.lactoferrin | Gal.d.1 |
| Hev.b.5 | Gad.c.1 | Gal.d.3 |
| Hev.b.6.01 | Gal.d.2 | Gly.m.5 |
| Tri.a.14 | Ole.e.7 | Jug.r.1 |
|  | Sal.k.1 | Par.j.2 |
|  | Ses.i.1 | Pla.a.1 |
|  | Tri.a.19.0101 | Pla.l.1 |
|  | Tri.a.aA_TI |  |
|  |  |  |
|  |  |  |

Table S4

|  |  |  |  |  |  |  |
| --- | --- | --- | --- | --- | --- | --- |
|  |  |  |  |  |  |  |
|  | **Age** | | | | | |
|  |  |  |  |  |  |  |
|  |  |  |  |  |  |  |
| **Component** | **1** | **3** | **5** | **8** | **11** | **16** |
|  |  |  |  |  |  |  |
|  |  |  |  |  |  |  |
| Bos.d.8 | 0 | **1** | *3* | **1** | **2** | 0 |
| Ber.e.1 | 0 | 0 | *4* | **1** | **1** | **1** |
| Cla.h.8 | 0 | 0 | **1** | *5* | 0 | **2** |
| Api.m.4 | 0 | 0 | **1** | *3* | 0 | **2** |
| Pen.m.4 | 0 | **1** | **2** | *10* | **1** | *3* |
| Jug.r.1 | 0 | **1** | **2** | *3* | *4* | **2** |
| Ani.s.3 | 0 | 0 | **1** | *3* | *7* | **2** |
| Gal.d.3 | **1** | **2** | *4* | **1** | *13* | **2** |
| Equ.c.3 | 0 | **1** | *3* | **1** | *5* | **2** |
| Bla.g.2 | 0 | **1** | *3* | **2** | 0 | *3* |
| Asp.f.3 | 0 | 0 | *3* | **2** | **1** | *4* |
| Par.j.2 | 0 | 0 | *3* | *3* | **1** | **2** |
| Fag.e.2 | 0 | **1** | *3* | *8* | 0 | 0 |
| Phl.p.7 | 0 | 0 | *4* | **2** | *9* | *8* |
| Ole.e.9 | 0 | **1** | *5* | **2** | *3* | *3* |
| Gly.m.6 | **2** | **1** | *5* | **2** | *4* | *4* |
| Fel.d.2 | 0 | **1** | *6* | **1** | *10* | *6* |
| Cry.j.1 | 0 | **2** | *5* | **1** | *12* | *15* |
| Api.g.1 | 0 | 0 | *7* | **2** | *7* | *10* |
| Gal.d.5 | 0 | 0 | *9* | *8* | 0 | *7* |
| Bos.d.6 | **1** | *3* | *3* | **1** | *4* | **2** |
| Gal.d.2 | **2** | *3* | *6* | *4* | **2** | **1** |
| Ana.o.2 | *3* | **1** | *8* | *5* | 0 | **1** |
| Gal.d.1 | *6* | *4* | *12* | **2** | *3* | **2** |
|  |  |  |  |  |  |  |
|  |  |  |  |  |  |  |

Table S5

|  |  |
| --- | --- |
|  |  |
| **k = 1** | |
| **Broad** | |
|  |  |
|  |  |
| **Component** | **Frequency** |
|  |  |
|  |  |
| Alt.a.1 | 11 |
| Ana.o.2 | 3 |
| Ara.h.1 | 5 |
| Can.f.1 | 7 |
| Der.f.1 | 3 |
| Der.p.2 | 3 |
| Fel.d.1 | 12 |
| Fel.d.4 | 3 |
| Gal.d.1 | 6 |
| Phl.p.4 | 5 |
|  |  |
|  |  |

Table S6

|  |  |  |  |  |  |  |
| --- | --- | --- | --- | --- | --- | --- |
|  |  |  |  |  |  |  |
|  |  |  | **k = 1** |  |  |  |
|  |  |  | **Broad** |  |  |  |
|  |  |  |  |  |  |  |
|  |  |  |  |  |  |  |
| **Component** | **Frequency** | **Assignment** |  | **Component** | **Frequency** | **Assignment** |
|  |  | **Probability** |  |  |  | **Probability** |
|  |  |  |  |  |  |  |
|  |  |  |  |  |  |  |
| Alt.a.1 | 18 | 1 |  | Equ.c.1 | 7 | 1 |
| Ara.h.1 | 6 | 1 |  | Fel.d.1 | 24 | 0.999 |
| Ara.h.2 | 8 | 1 |  | Fel.d.4 | 4 | 1 |
| Ara.h.6 | 7 | 1 |  | Gal.d.1 | 4 | 1 |
| Bos.d.6 | 3 | 1 |  | Gal.d.2 | 3 | 1 |
| Can.f.1 | 12 | 1 |  | Jug.r.2 | 3 | 1 |
| Can.f.2 | 4 | 1 |  | Mus.m.1 | 3 | 1 |
| Can.f.5 | 7 | 1 |  | Phl.p.4 | 20 | 0.992 |
| Cor.a.1.0401 | 5 | 1 |  | Phl.p.5 | 7 | 1 |
| Cup.a.1 | 3 | 1 |  | Pla.a.2 | 7 | 1 |
| Cyn.d.1 | 10 | 1 |  |  |  |  |
|  |  |  |  |  |  |  |
|  |  |  |  |  |  |  |
|  |  |  |  |  |  |  |
|  | **k = 2** |  |  |  | **k = 3** |  |
|  | **House Dust Mite** |  |  |  | **Grass** |  |
|  |  |  |  |  |  |  |
|  |  |  |  |  |  |  |
| **Component** | **Frequency** | **Assignment** |  | **Component** | **Frequency** | **Assignment** |
|  |  | **Probability** |  |  |  | **Probability** |
|  |  |  |  |  |  |  |
|  |  |  |  |  |  |  |
| Der.f.1 | 22 | 1 |  | Phl.p.1 | 29 | 0.999 |
| Der.f.2 | 19 | 1 |  |  |  |  |
| Der.p.1 | 27 | 1 |  |  |  |  |
| Der.p.2 | 19 | 1 |  |  |  |  |
|  |  |  |  |  |  |  |
|  |  |  |  |  |  |  |

Table S7

|  |  |  |  |  |  |  |  |  |  |  |
| --- | --- | --- | --- | --- | --- | --- | --- | --- | --- | --- |
|  |  |  |  |  |  |  |  |  |  |  |
|  |  |  |  |  | **k = 1** |  |  |  |  |  |
|  |  |  |  |  | **Broad** |  |  |  |  |  |
|  |  |  |  |  |  |  |  |  |  |  |
|  |  |  |  |  |  |  |  |  |  |  |
| **Component** | **Frequency** | **Assignment** |  | **Component** | **Frequency** | **Assignment** |  | **Component** | **Frequency** | **Assignment** |
|  |  | **Probability** |  |  |  | **Probability** |  |  |  | **Probability** |
|  |  |  |  |  |  |  |  |  |  |  |
|  |  |  |  |  |  |  |  |  |  |  |
| Act.d.1 | 4 | 1 |  | Can.f.3 | 6 | 1 |  | Lep.d.2 | 18 | 1 |
| Act.d.2 | 3 | 1 |  | Can.f.5 | 9 | 1 |  | Mal.d.1 | 9 | 1 |
| Aln.g.1 | 16 | 1 |  | Che.a.1 | 8 | 1 |  | Mer.a.1 | 4 | 1 |
| Ana.o.2 | 8 | 1 |  | Cor.a.1.0101 | 7 | 1 |  | Mus.m.1 | 6 | 1 |
| Api.g.1 | 7 | 1 |  | Cor.a.1.0401 | 32 | 1 |  | MUXF3 | 13 | 1 |
| Ara.h.1 | 14 | 1 |  | Cry.j.1 | 5 | 1 |  | Ole.e.1 | 10 | 1 |
| Ara.h.2 | 11 | 1 |  | Cup.a.1 | 10 | 1 |  | Ole.e.9 | 5 | 1 |
| Ara.h.3 | 4 | 1 |  | Der.p.10 | 3 | 1 |  | Par.j.2 | 3 | 1 |
| Ara.h.6 | 16 | 1 |  | Equ.c.1 | 20 | 1 |  | Pen.m.2 | 4 | 1 |
| Ara.h.8 | 4 | 1 |  | Equ.c.3 | 3 | 1 |  | Phl.p.11 | 8 | 1 |
| Asp.f.3 | 3 | 1 |  | Fag.e.2 | 3 | 1 |  | Phl.p.12 | 4 | 1 |
| Ber.e.1 | 4 | 1 |  | Fel.d.2 | 6 | 1 |  | Phl.p.2 | 27 | 1 |
| Bet.v.1 | 24 | 1 |  | Fel.d.4 | 18 | 1 |  | Phl.p.6 | 34 | 1 |
| Bet.v.2 | 5 | 1 |  | Gal.d.1 | 12 | 1 |  | Phl.p.7 | 4 | 1 |
| Bla.g.2 | 3 | 1 |  | Gal.d.2 | 6 | 1 |  | Pla.a.2 | 15 | 1 |
| Blo.t.5 | 5 | 1 |  | Gal.d.3 | 4 | 1 |  | Pru.p.1 | 7 | 1 |
| Bos.d.6 | 3 | 1 |  | Gal.d.5 | 9 | 1 |  | Pru.p.3 | 5 | 1 |
| Bos.d.8 | 3 | 1 |  | Gly.m.6 | 5 | 1 |  | Ves.v.5 | 11 | 1 |
| Can.f.1 | 25 | 1 |  | Hev.b.8 | 9 | 1 |  |  |  |  |
| Can.f.2 | 4 | 1 |  | Jug.r.2 | 17 | 1 |  |  |  |  |
|  |  |  |  |  |  |  |  |  |  |  |
|  |  |  |  |  |  |  |  |  |  |  |
|  |  |  |  |  |  |  |  |  |  |  |
|  | **k = 2** |  |  |  | **k = 3** |  |  |  | **k = 4** |  |
|  | **House Dust Mite** |  |  |  | **Grass/cat** |  |  |  | ***Alternaria*** |  |
|  |  |  |  |  |  |  |  |  |  |  |
|  |  |  |  |  |  |  |  |  |  |  |
| **Component** | **Frequency** | **Assignment** |  | **Component** | **Frequency** | **Assignment** |  | **Component** | **Frequency** | **Assignment** |
|  |  | **Probability** |  |  |  | **Probability** |  |  |  | **Probability** |
|  |  |  |  |  |  |  |  |  |  |  |
|  |  |  |  |  |  |  |  |  |  |  |
| Der.f.1 | 75 | 1 |  | Cyn.d.1 | 66 | 1 |  | Alt.a.1 | 72 | 1 |
| Der.f.2 | 72 | 1 |  | Fel.d.1 | 83 | 0.971 |  |  |  |  |
| Der.p.1 | 82 | 1 |  | Phl.p.1 | 104 | 1 |  |  |  |  |
| Der.p.2 | 73 | 1 |  | Phl.p.4 | 89 | 1 |  |  |  |  |
|  |  |  |  | Phl.p.5 | 71 | 1 |  |  |  |  |
|  |  |  |  |  |  |  |  |  |  |  |
|  |  |  |  |  |  |  |  |  |  |  |

Table S8

|  |  |  |  |  |  |  |  |  |  |  |
| --- | --- | --- | --- | --- | --- | --- | --- | --- | --- | --- |
|  |  |  |  |  |  |  |  |  |  |  |
|  |  |  |  |  | **k = 1** |  |  |  |  |  |
|  |  |  |  |  | **Broad** |  |  |  |  |  |
|  |  |  |  |  |  |  |  |  |  |  |
|  |  |  |  |  |  |  |  |  |  |  |
| **Component** | **Frequency** | **Assignment** |  | **Component** | **Frequency** | **Assignment** |  | **Component** | **Frequency** | **Assignment** |
|  |  | **Probability** |  |  |  | **Probability** |  |  |  | **Probability** |
|  |  |  |  |  |  |  |  |  |  |  |
|  |  |  |  |  |  |  |  |  |  |  |
| Act.d.1 | 4 | 1 |  | Can.f.3 | 5 | 1 |  | Mal.d.1 | 19 | 1 |
| Act.d.2 | 6 | 1 |  | Can.f.5 | 6 | 1 |  | Mer.a.1 | 9 | 1 |
| Act.d.8 | 3 | 1 |  | Che.a.1 | 3 | 1 |  | Mus.m.1 | 4 | 1 |
| Aln.g.1 | 23 | 1 |  | Cla.h.8 | 5 | 1 |  | MUXF3 | 5 | 1 |
| Ana.o.2 | 5 | 1 |  | Cor.a.1.0101 | 12 | 1 |  | Ole.e.1 | 32 | 1 |
| Ani.s.3 | 3 | 1 |  | Cor.a.1.0401 | 35 | 1 |  | Par.j.2 | 3 | 1 |
| Api.m.4 | 3 | 1 |  | Cup.a.1 | 7 | 1 |  | Pen.m.1 | 3 | 1 |
| Ara.h.1 | 8 | 1 |  | Der.p.10 | 4 | 1 |  | Pen.m.2 | 4 | 1 |
| Ara.h.2 | 10 | 1 |  | Equ.c.1 | 13 | 1 |  | Pen.m.4 | 10 | 1 |
| Ara.h.3 | 4 | 1 |  | Fag.e.2 | 8 | 1 |  | Phl.p.11 | 15 | 1 |
| Ara.h.6 | 11 | 1 |  | Fel.d.4 | 8 | 1 |  | Phl.p.12 | 9 | 1 |
| Ara.h.8 | 7 | 1 |  | Gal.d.2 | 4 | 1 |  | Phl.p.2 | 37 | 0.998 |
| Bet.v.1 | 39 | 1 |  | Gal.d.5 | 8 | 1 |  | Phl.p.6 | 38 | 0.994 |
| Bet.v.2 | 9 | 1 |  | Gly.m.4 | 3 | 1 |  | Pla.a.2 | 7 | 1 |
| Bla.g.7 | 3 | 1 |  | Hev.b.8 | 12 | 1 |  | Pru.p.1 | 9 | 1 |
| Blo.t.5 | 4 | 1 |  | Jug.r.1 | 3 | 1 |  | Pru.p.3 | 3 | 1 |
| Can.f.1 | 31 | 1 |  | Jug.r.2 | 8 | 1 |  | Ves.v.5 | 7 | 1 |
| Can.f.2 | 10 | 1 |  | Lep.d.2 | 18 | 1 |  |  |  |  |
|  |  |  |  |  |  |  |  |  |  |  |
|  |  |  |  |  |  |  |  |  |  |  |
|  |  |  |  |  |  |  |  |  |  |  |
|  | **k = 2** |  |  |  | **k = 3** |  |  |  | **k = 4** |  |
|  | **House Dust Mite** |  |  |  | **Grass/cat** |  |  |  | ***Alternaria*** |  |
|  |  |  |  |  |  |  |  |  |  |  |
|  |  |  |  |  |  |  |  |  |  |  |
| **Component** | **Frequency** | **Assignment** |  | **Component** | **Frequency** | **Assignment** |  | **Component** | **Frequency** | **Assignment** |
|  |  | **Probability** |  |  |  | **Probability** |  |  |  | **Probability** |
|  |  |  |  |  |  |  |  |  |  |  |
|  |  |  |  |  |  |  |  |  |  |  |
| Der.f.1 | 75 | 1 |  | Cyn.d.1 | 80 | 1 |  | Alt.a.1 | 63 | 1 |
| Der.f.2 | 73 | 1 |  | Fel.d.1 | 71 | 0.696 |  |  |  |  |
| Der.p.1 | 80 | 1 |  | Phl.p.1 | 118 | 1 |  |  |  |  |
| Der.p.2 | 77 | 1 |  | Phl.p.4 | 95 | 1 |  |  |  |  |
|  |  |  |  | Phl.p.5 | 87 | 1 |  |  |  |  |
|  |  |  |  |  |  |  |  |  |  |  |
|  |  |  |  |  |  |  |  |  |  |  |

Table S9

|  |  |  |  |  |  |  |  |  |  |  |
| --- | --- | --- | --- | --- | --- | --- | --- | --- | --- | --- |
|  |  |  |  |  |  |  |  |  |  |  |
|  |  |  |  |  | **k = 1** |  |  |  |  |  |
|  |  |  |  |  | **Broad** |  |  |  |  |  |
|  |  |  |  |  |  |  |  |  |  |  |
|  |  |  |  |  |  |  |  |  |  |  |
| **Component** | **Frequency** | **Assignment** |  | **Component** | **Frequency** | **Assignment** |  | **Component** | **Frequency** | **Assignment** |
|  |  | **Probability** |  |  |  | **Probability** |  |  |  | **Probability** |
|  |  |  |  |  |  |  |  |  |  |  |
|  |  |  |  |  |  |  |  |  |  |  |
| Act.d.1 | 5 | 1 |  | Blo.t.5 | 9 | 1 |  | Gly.m.4 | 14 | 1 |
| Act.d.2 | 7 | 1 |  | Bos.d.6 | 4 | 1 |  | Gly.m.6 | 4 | 1 |
| Act.d.8 | 3 | 1 |  | Can.f.1 | 37 | 1 |  | Jug.r.1 | 4 | 1 |
| Alt.a.1 | 21 | 1 |  | Can.f.2 | 9 | 1 |  | Jug.r.2 | 16 | 1 |
| Alt.a.6 | 3 | 1 |  | Can.f.3 | 8 | 1 |  | Jug.r.3 | 3 | 1 |
| Ani.s.3 | 7 | 1 |  | Can.f.5 | 23 | 1 |  | Lep.d.2 | 35 | 1 |
| Api.g.1 | 7 | 1 |  | Che.a.1 | 16 | 1 |  | Mus.m.1 | 11 | 1 |
| Ara.h.1 | 14 | 1 |  | Cry.j.1 | 12 | 1 |  | Ole.e.9 | 3 | 1 |
| Ara.h.2 | 20 | 1 |  | Cup.a.1 | 26 | 0.870 |  | Pen.m.1 | 7 | 1 |
| Ara.h.3 | 9 | 1 |  | Der.p.10 | 7 | 1 |  | Pen.m.2 | 5 | 1 |
| Ara.h.6 | 19 | 1 |  | Equ.c.1 | 19 | 1 |  | Phl.p.7 | 9 | 1 |
| Ara.h.8 | 19 | 0.817 |  | Equ.c.3 | 5 | 1 |  | Pla.a.2 | 15 | 1 |
| Art.v.1 | 4 | 1 |  | Fel.d.2 | 10 | 1 |  | Pol.d.5 | 3 | 1 |
| Asp.f.6 | 3 | 1 |  | Fel.d.4 | 20 | 1 |  | Pru.p.3 | 4 | 1 |
| Bet.v.4 | 5 | 1 |  | Gal.d.1 | 3 | 1 |  | Ves.v.5 | 5 | 1 |
| Bla.g.7 | 7 | 1 |  | Gal.d.3 | 13 | 1 |  |  |  |  |
|  |  |  |  |  |  |  |  |  |  |  |
|  |  |  |  |  |  |  |  |  |  |  |
|  |  |  |  |  |  |  |  |  |  |  |
|  | **k = 2** |  |  |  | **k = 3** |  |  |  | **k = 4** |  |
|  | **House Dust Mite** |  |  |  | **Grass** |  |  |  | **Cat** |  |
|  |  |  |  |  |  |  |  |  |  |  |
|  |  |  |  |  |  |  |  |  |  |  |
| **Component** | **Frequency** | **Assignment** |  | **Component** | **Frequency** | **Assignment** |  | **Component** | **Frequency** | **Assignment** |
|  |  | **Probability** |  |  |  | **Probability** |  |  |  | **Probability** |
|  |  |  |  |  |  |  |  |  |  |  |
|  |  |  |  |  |  |  |  |  |  |  |
| Der.f.1 | 92 | 1 |  | Cyn.d.1 | 115 | 1 |  | Fel.d.1 | 80 | 1 |
| Der.f.2 | 99 | 1 |  | Phl.p.1 | 150 | 1 |  |  |  |  |
| Der.p.1 | 97 | 1 |  | Phl.p.2 | 83 | 1 |  |  |  |  |
| Der.p.2 | 95 | 1 |  | Phl.p.4 | 106 | 1 |  |  |  |  |
|  |  |  |  | Phl.p.5 | 125 | 1 |  |  |  |  |
|  |  |  |  | Phl.p.6 | 74 | 1 |  |  |  |  |
|  |  |  |  |  |  |  |  |  |  |  |
|  |  |  |  |  |  |  |  |  |  |  |
|  |  |  |  |  |  |  |  |  |  |  |
|  |  |  |  |  | **k = 5** |  |  |  |  |  |
|  |  |  |  |  | **PR-10/profilin** |  |  |  |  |  |
|  |  |  |  |  |  |  |  |  |  |  |
|  |  |  |  |  |  |  |  |  |  |  |
|  |  |  |  | **Component** | **Frequency** | **Assignment** |  |  |  |  |
|  |  |  |  |  |  | **Probability** |  |  |  |  |
|  |  |  |  |  |  |  |  |  |  |  |
|  |  |  |  |  |  |  |  |  |  |  |
|  |  |  |  | Aln.g.1 | 43 | 1 |  |  |  |  |
|  |  |  |  | Bet.v.1 | 70 | 1 |  |  |  |  |
|  |  |  |  | Bet.v.2 | 32 | 1 |  |  |  |  |
|  |  |  |  | Cor.a.1.0101 | 30 | 1 |  |  |  |  |
|  |  |  |  | Cor.a.1.0401 | 49 | 1 |  |  |  |  |
|  |  |  |  | Hev.b.8 | 38 | 1 |  |  |  |  |
|  |  |  |  | Mal.d.1 | 39 | 1 |  |  |  |  |
|  |  |  |  | Mer.a.1 | 38 | 1 |  |  |  |  |
|  |  |  |  | MUXF3 | 27 | 0.808 |  |  |  |  |
|  |  |  |  | Ole.e.1 | 54 | 1 |  |  |  |  |
|  |  |  |  | Phl.p.11 | 46 | 1 |  |  |  |  |
|  |  |  |  | Phl.p.12 | 25 | 1 |  |  |  |  |
|  |  |  |  | Pru.p.1 | 36 | 1 |  |  |  |  |
|  |  |  |  |  |  |  |  |  |  |  |
|  |  |  |  |  |  |  |  |  |  |  |

Table S10

|  |  |  |  |  |  |  |  |  |  |  |
| --- | --- | --- | --- | --- | --- | --- | --- | --- | --- | --- |
|  |  |  |  |  |  |  |  |  |  |  |
|  |  |  |  |  | **k = 1** |  |  |  |  |  |
|  |  |  |  |  | **Broad** |  |  |  |  |  |
|  |  |  |  |  |  |  |  |  |  |  |
|  |  |  |  |  |  |  |  |  |  |  |
| **Component** | **Frequency** | **Assignment** |  | **Component** | **Frequency** | **Assignment** |  | **Component** | **Frequency** | **Assignment** |
|  |  | **Probability** |  |  |  | **Probability** |  |  |  | **Probability** |
|  |  |  |  |  |  |  |  |  |  |  |
|  |  |  |  |  |  |  |  |  |  |  |
| Act.d.1 | 4 | 1 |  | Bla.g.7 | 3 | 1 |  | Gly.m.6 | 4 | 1 |
| Act.d.2 | 7 | 1 |  | Blo.t.5 | 13 | 1 |  | Jug.r.2 | 13 | 1 |
| Act.d.8 | 15 | 1 |  | Can.f.1 | 38 | 1 |  | Jug.r.3 | 5 | 1 |
| Alt.a.1 | 24 | 1 |  | Can.f.2 | 9 | 1 |  | Lep.d.2 | 30 | 1 |
| Alt.a.6 | 4 | 1 |  | Can.f.3 | 8 | 1 |  | Mus.m.1 | 5 | 1 |
| Api.g.1 | 10 | 1 |  | Can.f.5 | 32 | 1 |  | Ole.e.9 | 3 | 1 |
| Ara.h.1 | 8 | 1 |  | Che.a.1 | 4 | 1 |  | Pen.m.1 | 6 | 1 |
| Ara.h.2 | 11 | 1 |  | Cor.a.8 | 4 | 1 |  | Pen.m.2 | 5 | 1 |
| Ara.h.3 | 8 | 1 |  | Cry.j.1 | 15 | 1 |  | Pen.m.4 | 3 | 1 |
| Ara.h.6 | 12 | 1 |  | Cup.a.1 | 20 | 1 |  | Phl.p.7 | 8 | 1 |
| Ara.h.9 | 4 | 1 |  | Der.p.10 | 6 | 1 |  | Pla.a.2 | 16 | 1 |
| Art.v.1 | 7 | 1 |  | Equ.c.1 | 24 | 1 |  | Pla.a.3 | 4 | 1 |
| Asp.f.3 | 4 | 1 |  | Fel.d.2 | 6 | 1 |  | Pol.d.5 | 3 | 1 |
| Asp.f.6 | 8 | 1 |  | Fel.d.4 | 22 | 1 |  | Pru.p.3 | 4 | 1 |
| Bet.v.4 | 5 | 1 |  | Gal.d.5 | 7 | 1 |  | Ves.v.5 | 4 | 1 |
| Bla.g.2 | 3 | 1 |  | Gly.m.4 | 16 | 1 |  |  |  |  |
|  |  |  |  |  |  |  |  |  |  |  |
|  |  |  |  |  |  |  |  |  |  |  |
|  |  |  |  |  |  |  |  |  |  |  |
|  | **k = 2** |  |  |  | **k = 3** |  |  |  | **k = 4** |  |
|  | **House Dust Mite** |  |  |  | **Grass** |  |  |  | **Cat** |  |
|  |  |  |  |  |  |  |  |  |  |  |
|  |  |  |  |  |  |  |  |  |  |  |
| **Component** | **Frequency** | **Assignment** |  | **Component** | **Frequency** | **Assignment** |  | **Component** | **Frequency** | **Assignment** |
|  |  | **Probability** |  |  |  | **Probability** |  |  |  | **Probability** |
|  |  |  |  |  |  |  |  |  |  |  |
|  |  |  |  |  |  |  |  |  |  |  |
| Der.f.1 | 84 | 1 |  | Cyn.d.1 | 109 | 1 |  | Fel.d.1 | 84 | 0.985 |
| Der.f.2 | 79 | 1 |  | Phl.p.1 | 154 | 1 |  |  |  |  |
| Der.p.1 | 87 | 1 |  | Phl.p.2 | 85 | 1 |  |  |  |  |
| Der.p.2 | 87 | 1 |  | Phl.p.4 | 95 | 1 |  |  |  |  |
|  |  |  |  | Phl.p.5 | 128 | 1 |  |  |  |  |
|  |  |  |  | Phl.p.6 | 91 | 1 |  |  |  |  |
|  |  |  |  |  |  |  |  |  |  |  |
|  |  |  |  |  |  |  |  |  |  |  |
|  |  |  |  |  |  |  |  |  |  |  |
|  |  |  | **k = 5** |  |  |  | **k = 6** |  |  |  |
|  |  |  | **PR-10** |  |  |  | **Profilin** |  |  |  |
|  |  |  |  |  |  |  |  |  |  |  |
|  |  |  |  |  |  |  |  |  |  |  |
|  |  | **Component** | **Frequency** | **Assignment** |  | **Component** | **Frequency** | **Assignment** |  |  |
|  |  |  |  | **Probability** |  |  |  | **Probability** |  |  |
|  |  |  |  |  |  |  |  |  |  |  |
|  |  |  |  |  |  |  |  |  |  |  |
|  |  | Aln.g.1 | 57 | 1 |  | Bet.v.2 | 41 | 1 |  |  |
|  |  | Ara.h.8 | 39 | 1 |  | Hev.b.8 | 49 | 1 |  |  |
|  |  | Bet.v.1 | 86 | 1 |  | Mer.a.1 | 50 | 1 |  |  |
|  |  | Cor.a.1.0101 | 45 | 1 |  | MUXF3 | 34 | 0.798 |  |  |
|  |  | Cor.a.1.0401 | 73 | 1 |  | Phl.p.11 | 41 | 0.798 |  |  |
|  |  | Mal.d.1 | 65 | 1 |  | Phl.p.12 | 41 | 1 |  |  |
|  |  | Ole.e.1 | 68 | 1 |  |  |  |  |  |  |
|  |  | Pru.p.1 | 49 | 1 |  |  |  |  |  |  |
|  |  |  |  |  |  |  |  |  |  |  |
|  |  |  |  |  |  |  |  |  |  |  |

Table S11

|  |
| --- |
|  |
| **Component** |
|  |
|  |
| Act.d.1 |
| Act.d.2 |
| Ara.h.1 |
| Ara.h.2 |
| Ara.h.3 |
| Ara.h.6 |
| Blo.t.5 |
| Can.f.1 |
| Can.f.2 |
| Can.f.3 |
| Can.f.5 |
| Che.a.1 |
| Cup.a.1 |
| Der.p.10 |
| Equ.c.1 |
| Fel.d.4 |
| Gal.d.1 |
| Jug.r.2 |
| Lep.d.2 |
| Mus.m.1 |
| Pen.m.2 |
| Pla.a.2 |
| Pru.p.3 |
| Ves.v.5 |
|  |
|  |

Table S12a

|  |  |  |  |  |  |  |  |
| --- | --- | --- | --- | --- | --- | --- | --- |
|  |  |  |  |  |  |  |  |
| **Frequency** |  |  | **Broad** | **Alternaria** | **Grass** | **HDM** | |
|  |  |  |  |  |  |  |  |
|  |  |  |  |  |  |  |  |
| 332 |  |  | 0 | 0 | 0 | 0 |  |
| 42 |  |  | 0 | 0 | 1 | 0 |  |
| 37 |  |  | 1 | 0 | 1 | 1 |  |
| 35 |  |  | 1 | 0 | 1 | 0 |  |
| 25 |  |  | 0 | 1 | 0 | 0 |  |
| 23 |  |  | 0 | 0 | 1 | 1 |  |
| 20 |  |  | 0 | 0 | 0 | 1 |  |
| 17 |  |  | 1 | 0 | 0 | 0 |  |
| 12 |  |  | 1 | 1 | 1 | 1 |  |
| 11 |  |  | 1 | 1 | 0 | 0 |  |
| 10 |  |  | 1 | 1 | 1 | 0 |  |
| 5 |  |  | 0 | 1 | 1 | 0 |  |
| 5 |  |  | 1 | 0 | 0 | 1 |  |
| 4 |  |  | 1 | 1 | 0 | 1 |  |
| 2 |  |  | 0 | 1 | 0 | 1 |  |
| 2 |  |  | 0 | 1 | 1 | 1 |  |
|  |  |  |  |  |  |  |  |
|  |  |  |  |  |  |  |  |

Table S12b

|  |  |  |  |  |  |  |  |  |  |
| --- | --- | --- | --- | --- | --- | --- | --- | --- | --- |
|  |  |  |  |  |  |  |  |  |  |
| **Frequency** |  |  | **Broad** | **PR-10** | **Profilin** | **Grass** | **Cat** | **HDM** | |
|  |  |  |  |  |  |  |  |  |  |
|  |  |  |  |  |  |  |  |  |  |
| 154 |  |  | 0 | 0 | 0 | 0 | 0 | 0 |  |
| 17 |  |  | 1 | 1 | 1 | 1 | 1 | 1 |  |
| 14 |  |  | 0 | 1 | 1 | 1 | 1 | 1 |  |
| 12 |  |  | 1 | 1 | 1 | 0 | 1 | 1 |  |
| 10 |  |  | 0 | 0 | 0 | 0 | 1 | 0 |  |
| 10 |  |  | 1 | 0 | 0 | 0 | 1 | 0 |  |
| 9 |  |  | 1 | 0 | 0 | 0 | 1 | 1 |  |
| 9 |  |  | 1 | 1 | 0 | 0 | 1 | 1 |  |
| 9 |  |  | 0 | 0 | 1 | 0 | 0 | 0 |  |
| 8 |  |  | 1 | 1 | 0 | 1 | 1 | 1 |  |
| 7 |  |  | 0 | 0 | 0 | 0 | 0 | 1 |  |
| 7 |  |  | 0 | 0 | 1 | 0 | 1 | 0 |  |
| 7 |  |  | 0 | 1 | 1 | 0 | 1 | 1 |  |
| 6 |  |  | 0 | 0 | 0 | 0 | 1 | 1 |  |
| 6 |  |  | 1 | 1 | 0 | 0 | 1 | 0 |  |
| 6 |  |  | 0 | 1 | 0 | 1 | 1 | 1 |  |
| 6 |  |  | 0 | 1 | 1 | 1 | 1 | 0 |  |
| 5 |  |  | 0 | 1 | 0 | 0 | 0 | 0 |  |
| 5 |  |  | 0 | 0 | 1 | 1 | 1 | 1 |  |
| 4 |  |  | 0 | 1 | 0 | 0 | 1 | 0 |  |
| 4 |  |  | 0 | 0 | 0 | 1 | 0 | 0 |  |
| 4 |  |  | 1 | 1 | 0 | 1 | 1 | 0 |  |
| 4 |  |  | 0 | 0 | 1 | 0 | 1 | 1 |  |
| 4 |  |  | 1 | 0 | 1 | 1 | 1 | 1 |  |
| 3 |  |  | 0 | 1 | 0 | 0 | 1 | 1 |  |
| 3 |  |  | 0 | 0 | 0 | 1 | 1 | 1 |  |
| 3 |  |  | 0 | 0 | 1 | 0 | 0 | 1 |  |
| 3 |  |  | 1 | 0 | 1 | 0 | 1 | 0 |  |
| 3 |  |  | 1 | 1 | 1 | 0 | 1 | 0 |  |
| 3 |  |  | 0 | 1 | 1 | 1 | 0 | 1 |  |
| 2 |  |  | 0 | 1 | 0 | 0 | 0 | 1 |  |
| 2 |  |  | 1 | 0 | 1 | 0 | 1 | 1 |  |
| 2 |  |  | 0 | 0 | 1 | 1 | 0 | 0 |  |
| 2 |  |  | 0 | 0 | 1 | 1 | 0 | 1 |  |
| 1 |  |  | 0 | 0 | 0 | 1 | 0 | 1 |  |
| 1 |  |  | 0 | 0 | 0 | 1 | 1 | 0 |  |
| 1 |  |  | 0 | 1 | 0 | 1 | 1 | 0 |  |
| 1 |  |  | 1 | 0 | 0 | 1 | 1 | 1 |  |
| 1 |  |  | 0 | 1 | 1 | 0 | 0 | 1 |  |
| 1 |  |  | 0 | 1 | 1 | 0 | 1 | 0 |  |
| 1 |  |  | 0 | 0 | 1 | 1 | 1 | 0 |  |
| 1 |  |  | 1 | 0 | 1 | 1 | 1 | 0 |  |
|  |  |  |  |  |  |  |  |  |  |
|  |  |  |  |  |  |  |  |  |  |

Table S13

|  |  |  |  |  |  |  |  |  |  |  |  |
| --- | --- | --- | --- | --- | --- | --- | --- | --- | --- | --- | --- |
|  |  |  |  |  |  |  |  |  |  |  |  |
| **Age 5** |  |  | **Age 16 Clusters** | | | | | |  |  |  |
|  |  |  |  |  |  |  |  |  |  |  |  |
| **Clusters** |  |  |  |  |  |  |  |  |  |  |  |
|  |  |  | **Broad** | **HDM** | **Grass** | **Cat** | **PR-10** | **Profilin** |  |  | **Total** |
|  |  |  |  |  |  |  |  |  |  |  |  |
|  |  |  |  |  |  |  |  |  |  |  |  |
| **Broad** |  |  | 51 | 37 | 53 | 32 | 46 | 30 |  |  | 62 |
| **HDM** |  |  | 34 | 44 | 37 | 26 | 31 | 20 |  |  | 47 |
| **Grass/cat** |  |  | 58 | 41 | 71 | 39 | 53 | 41 |  |  | 78 |
| ***Alternaria*** |  |  | 14 | 14 | 16 | 5 | 11 | 10 |  |  | 27 |
|  |  |  |  |  |  |  |  |  |  |  |  |
|  |  |  |  |  |  |  |  |  |  |  |  |
| **Total** |  |  | 92 | 79 | 121 | 58 | 82 | 64 |  |  |  |
|  |  |  |  |  |  |  |  |  |  |  |  |
|  |  |  |  |  |  |  |  |  |  |  |  |

Table S14

|  |  |  |  |  |  |  |
| --- | --- | --- | --- | --- | --- | --- |
|  |  |  |  |  |  |  |
|  |  |  | **Cluster Responses** | |  |  |
|  |  |  |  |  |  |  |
|  |  |  |  |  |  |  |
|  |  |  | **Age 5's** | **Age 16's** |  |  |
|  |  |  |  |  |  |  |
|  |  |  |  |  |  |  |
|  |  | Current Asthma | 0.73 | 0.76 |  |  |
|  |  | Current Wheeze | 0.63 | 0.70 |  |  |
|  |  | Current Rhinitis | 0.65 | 0.81 |  |  |
|  |  |  |  |  |  |  |
|  |  |  |  |  |  |  |
