# Supplementary figures and images for: Evolution of IgE responses to multiple allergen components throughout childhood
Source: J Allergy Clin Immunol. 2018 Oct;142(4):1322–30. doi: 10.1016/j.jaci.2017.11.064 (PMC6170973; doi:10.1016/j.jaci.2017.11.064)

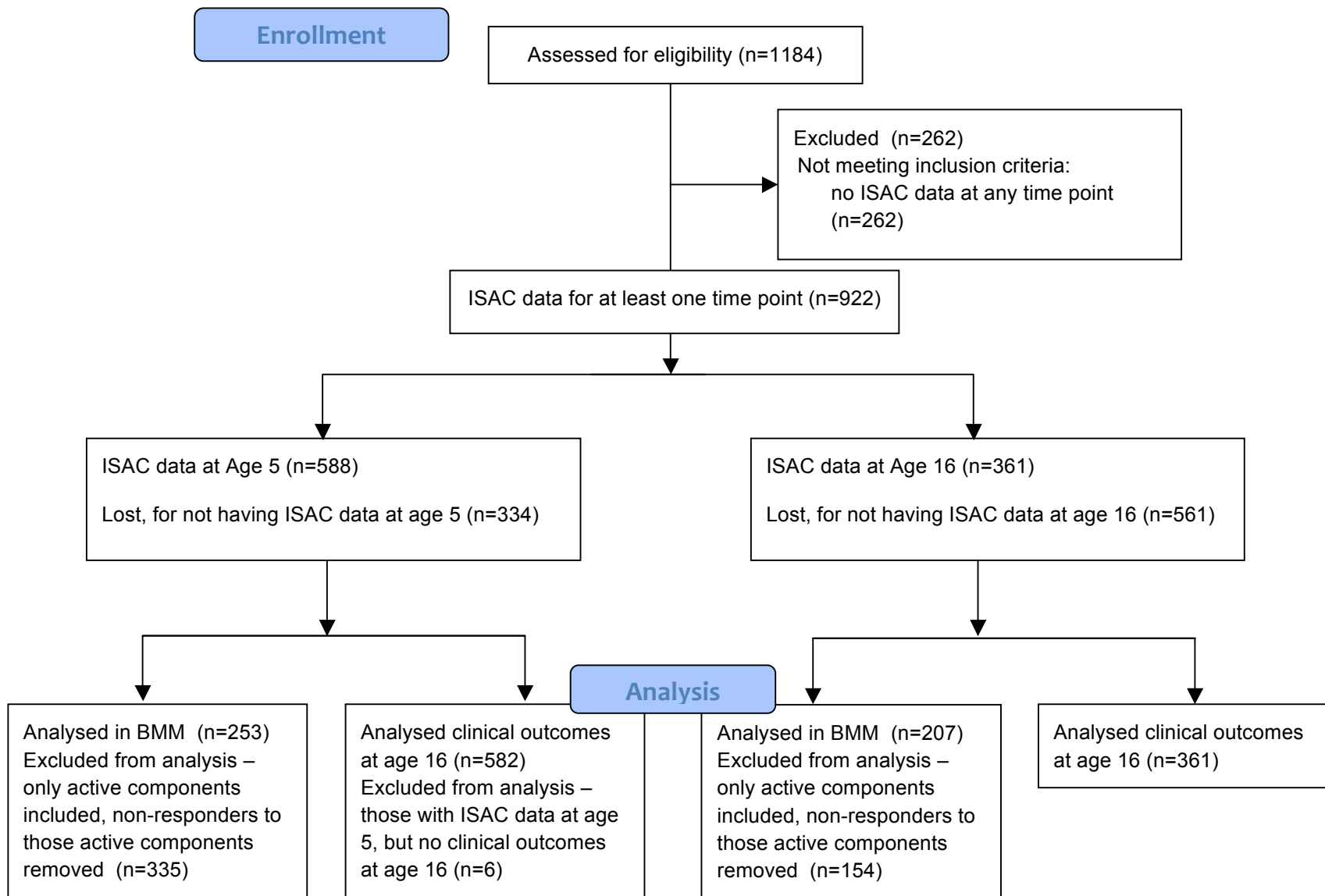

Supplement: Fig E1 [file mmc3.pdf]

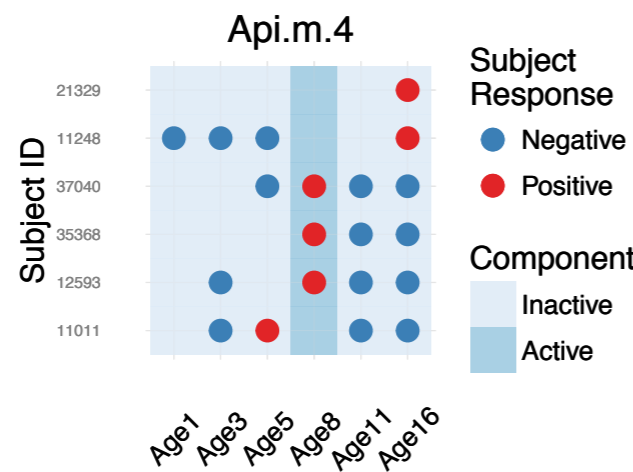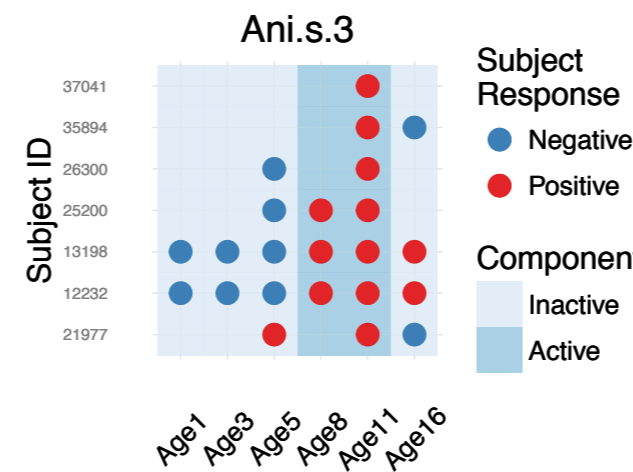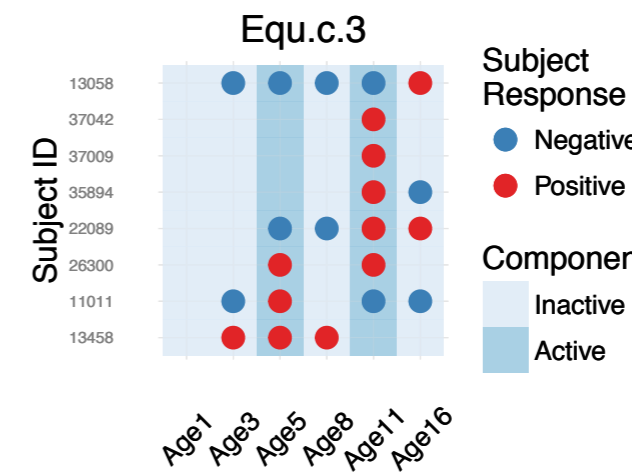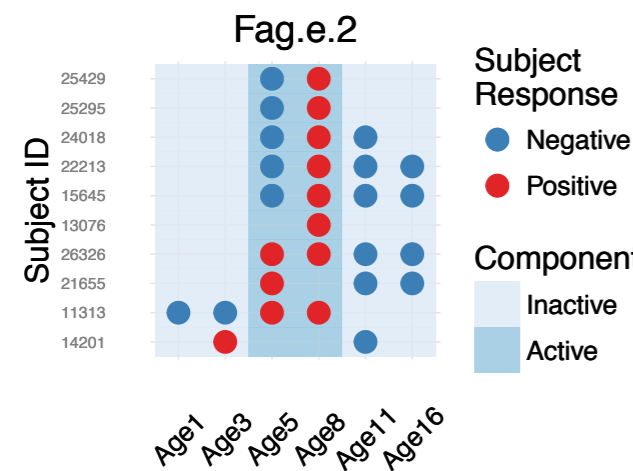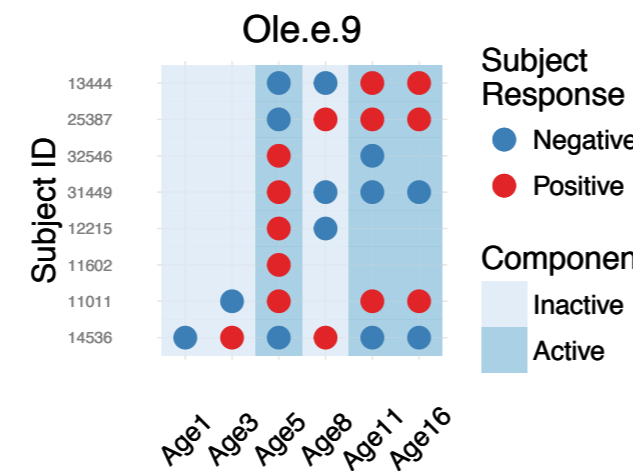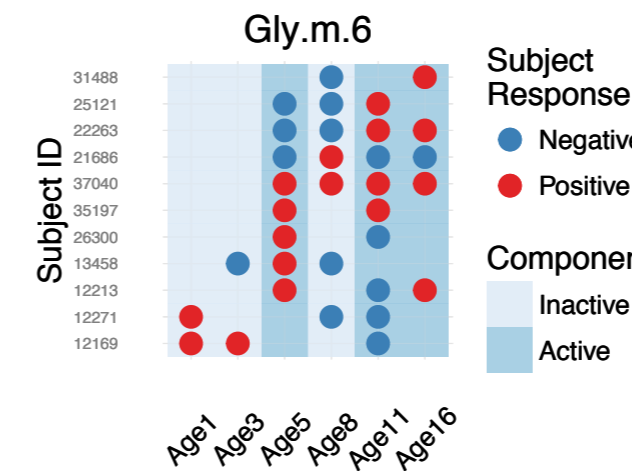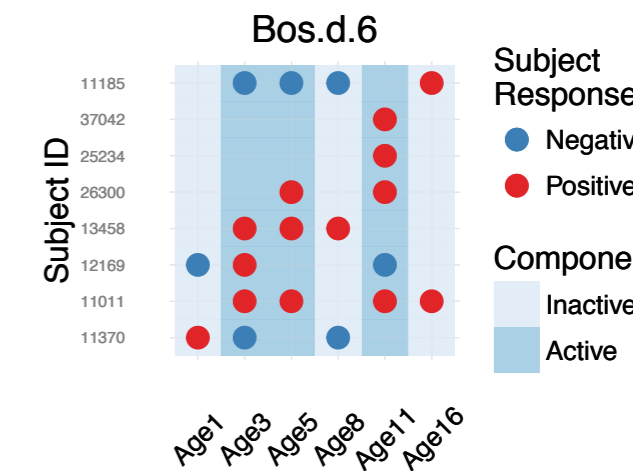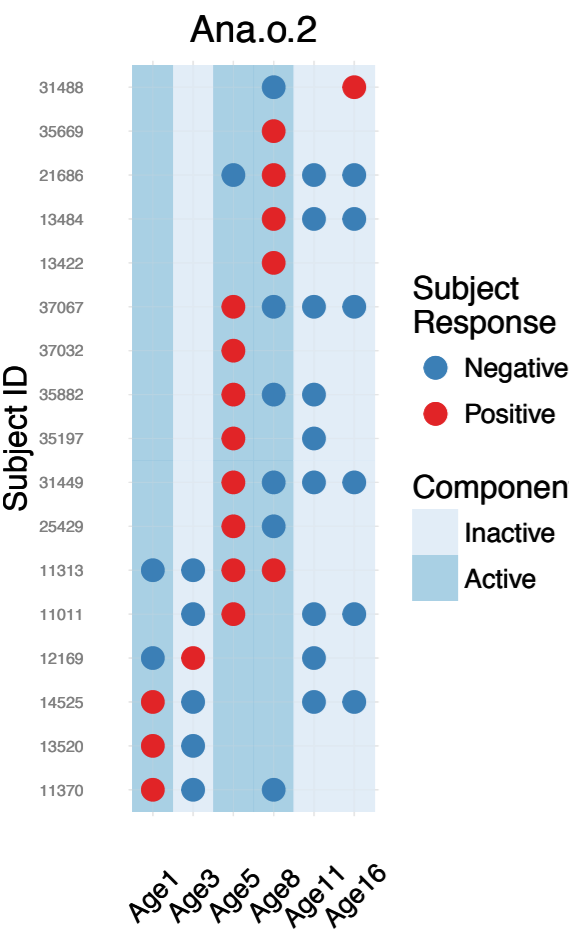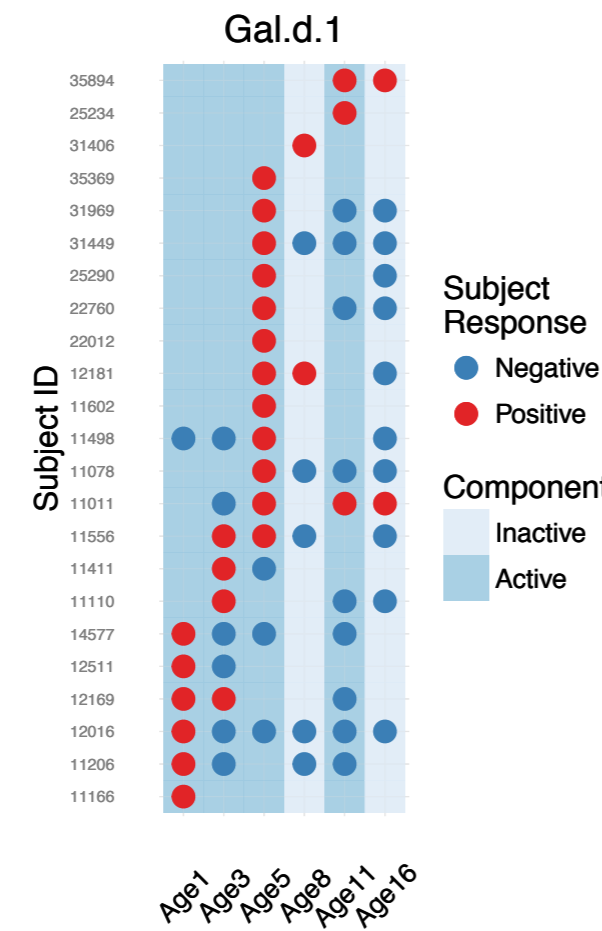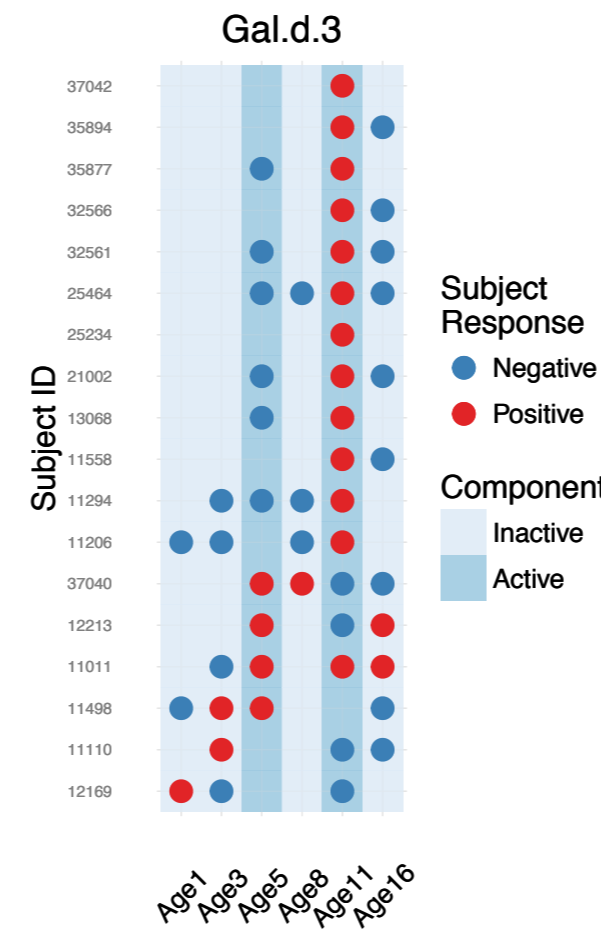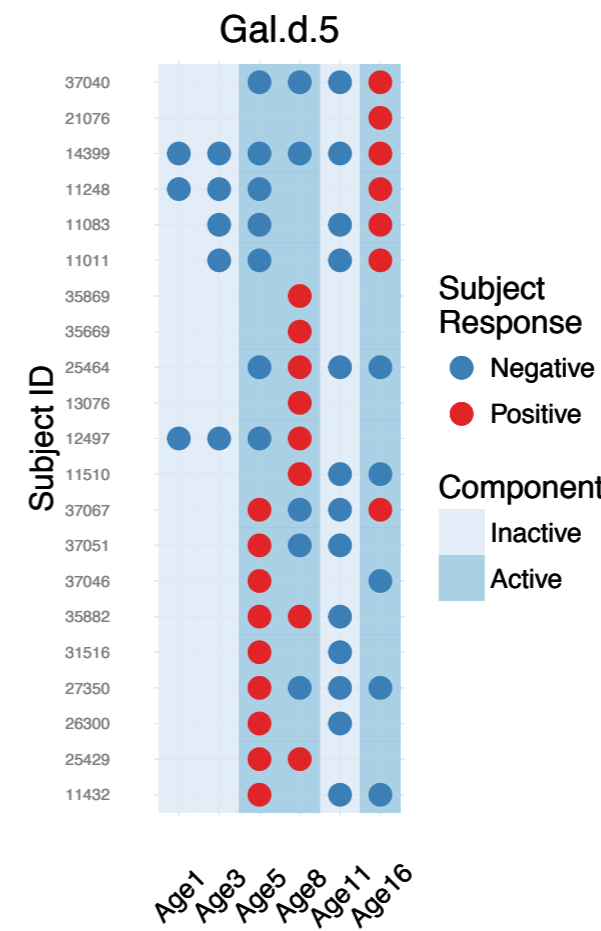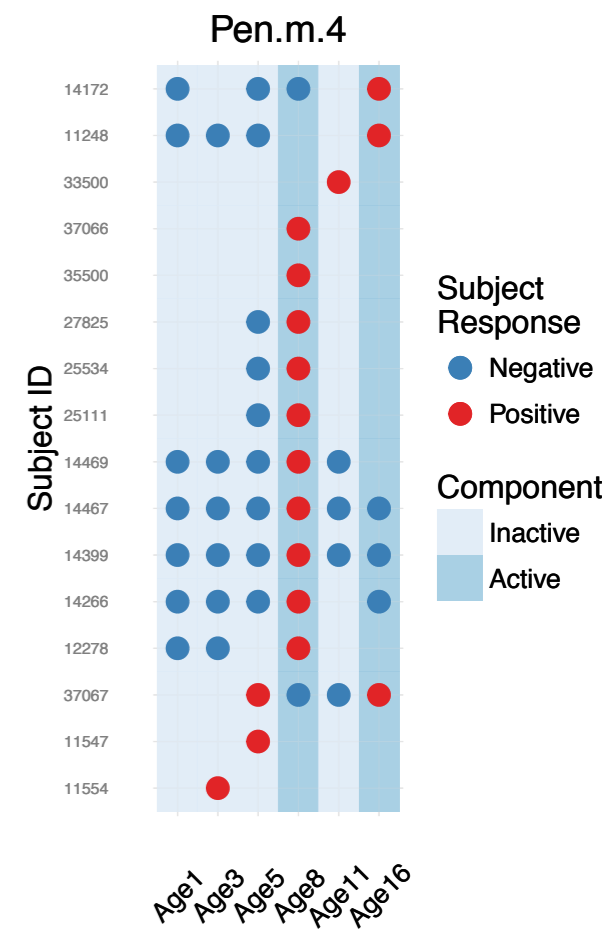

Supplement: Fig E2A [file mmc4.pdf]

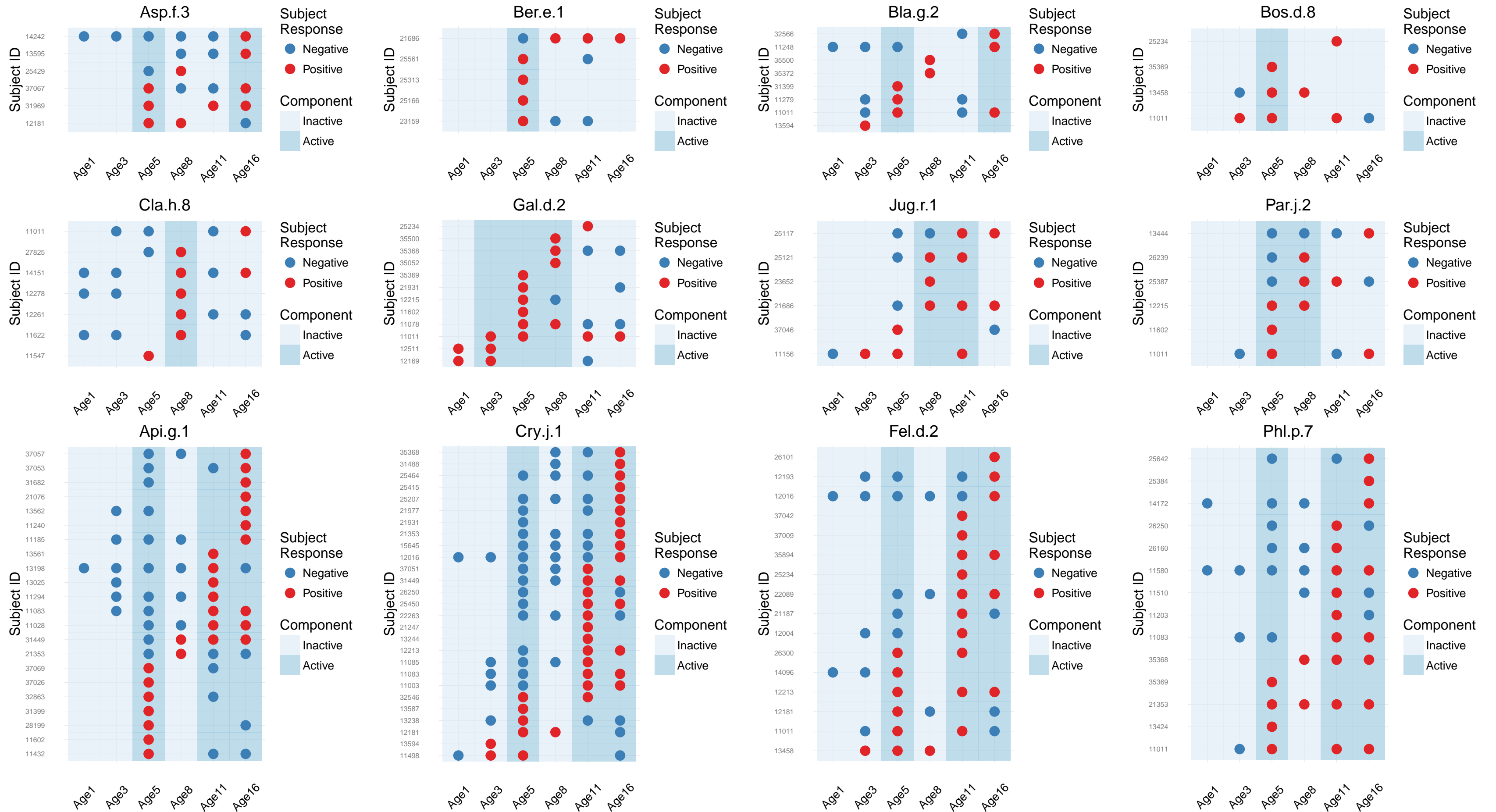

Supplement: Fig E2B [file mmc5.pdf]

**(a)****Current Asthma**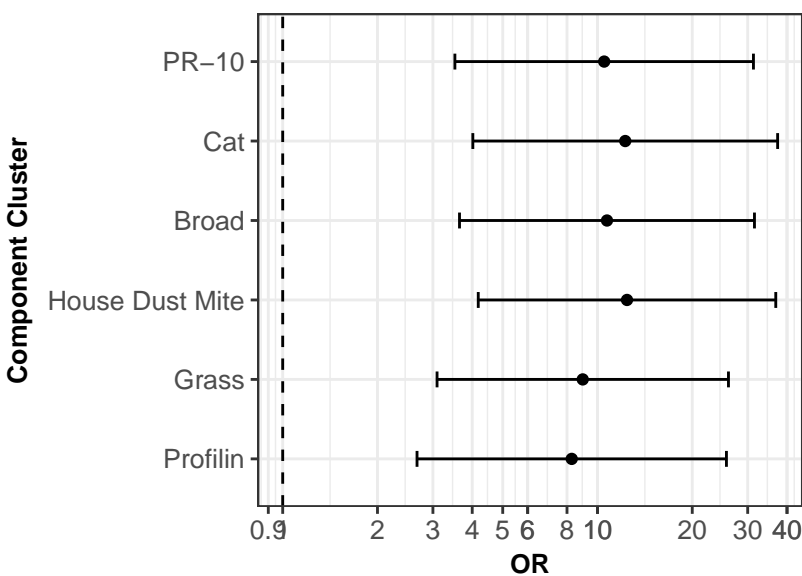**Current Wheeze**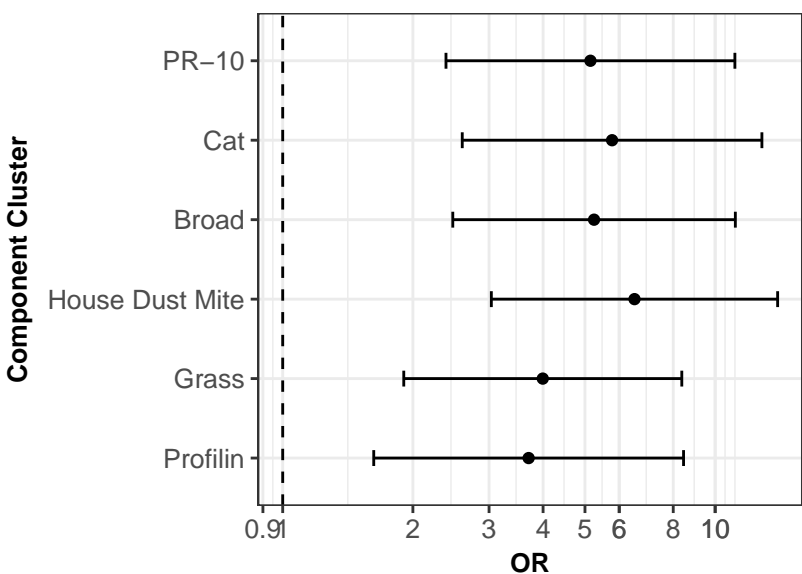**Current Rhinitis**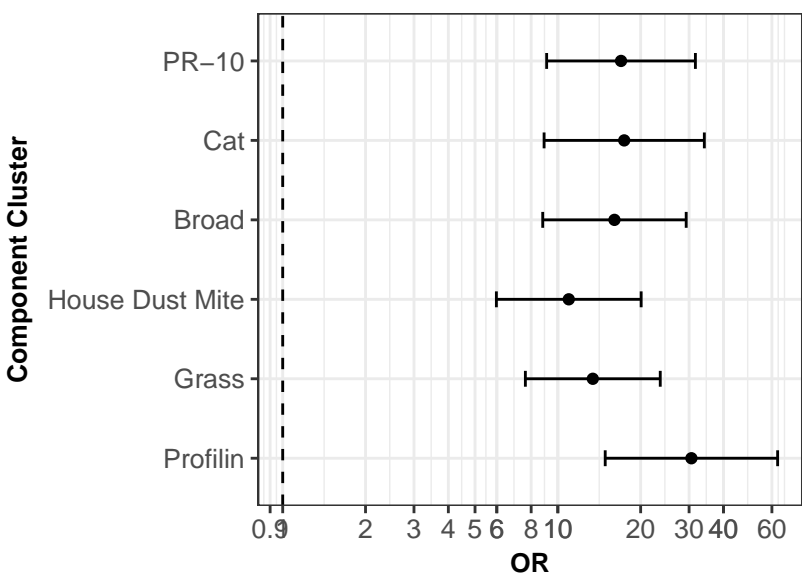

Supplement: Fig E3A [file mmc6.pdf]

**(b)****Current Asthma**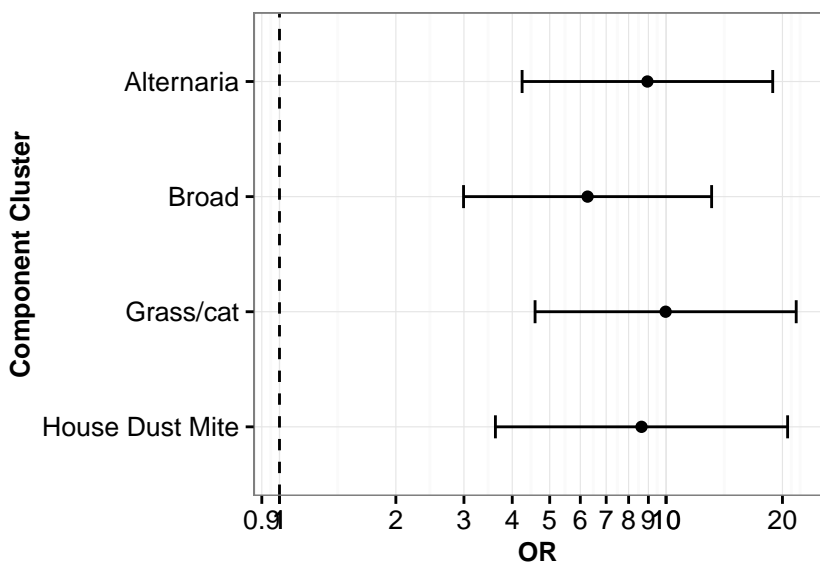**Current Wheeze**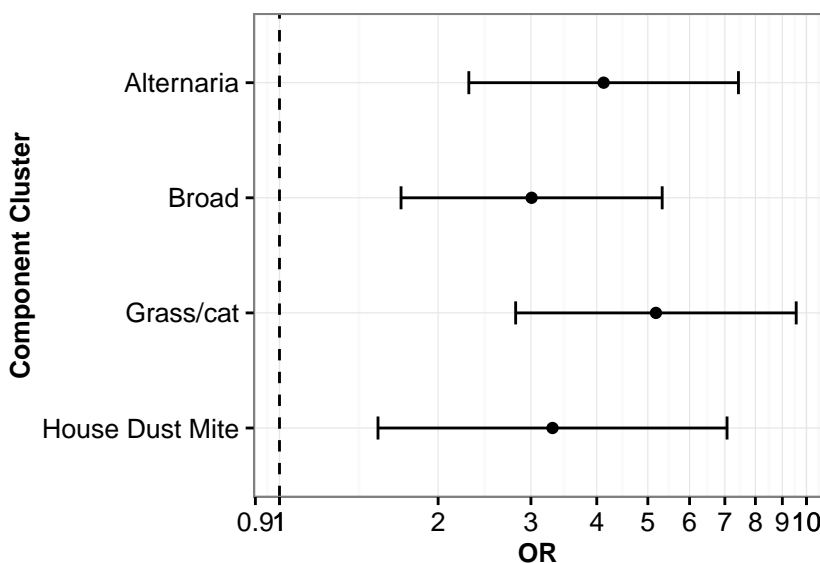**Current Rhinitis**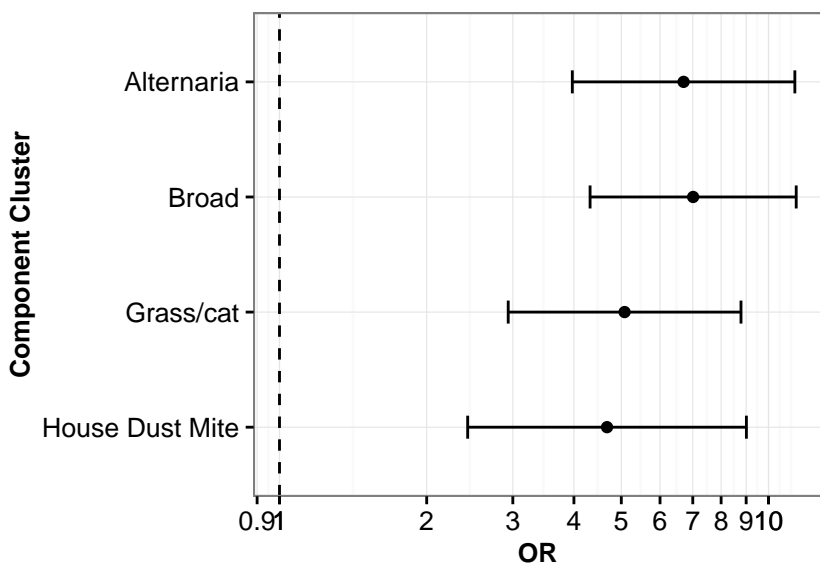

Supplement: Fig E3B [file mmc7.pdf]
